# Supplementary material for: Neural tube patterning: from a minimal model for rostrocaudal patterning toward an integrated 3D model
Source: iScience. 2021 May 20;24(6):102559. doi: 10.1016/j.isci.2021.102559 (PMC8184516; doi:10.1016/j.isci.2021.102559)
Supplement: Document S1. Figures S1–S5 [file mmc1.pdf]

iScience, Volume *24*

Supplemental information

Neural tube patterning: from a minimal model for rostrocaudal patterning toward an integrated 3D model

Max Brambach, Ariane Ernst, Sara Nolbrant, Janelle Drouin-Ouellet, Agnete Kirkeby, Malin Parmar, and Victor Olariu

Figure S1. Performance of the rostrocaudal model topology, Related to Figure 1

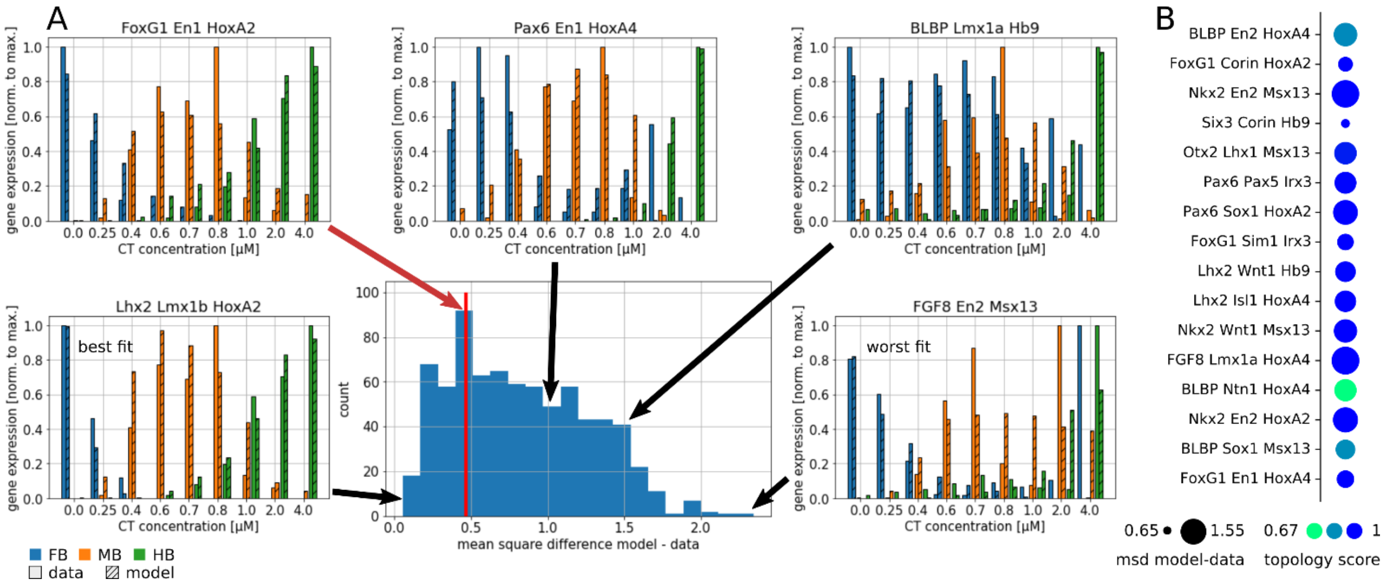

Figure S1: Performance of the rostrocaudal model topology. **A**: The rostrocaudal model was optimised towards all permutations of region-specific genes using the tristable topology (Figure 1 E). The histogram shows the distribution of mean squared differences (msd) between the data and the optimised model outputs. The red line indicates the gene selection used for further analysis (FoxG1, En1, HoxA2). The bar plots show examples of the detailed model performance for different msd values as indicated by the arrows. **B**: 16 gene combinations were randomly selected, and the optimal model topology was determined using the unbiased topology selection process. The similarity of the optimal model topology to the one found for (FoxG1, En1, HoxA2) is expressed using a topology score which is defined as the number of identical connections (activation or repression) normalised to the number of varied connections; 1=all connections are identical to the ones found for (FoxG1, EN1, HoxA2), 0=no connections match. Note that most optimal topologies are very similar or identical to the one used for (FoxG1, En1, HoxA2).

Figure S2. U-node parameter influence on patterning results, Related to Figure 3

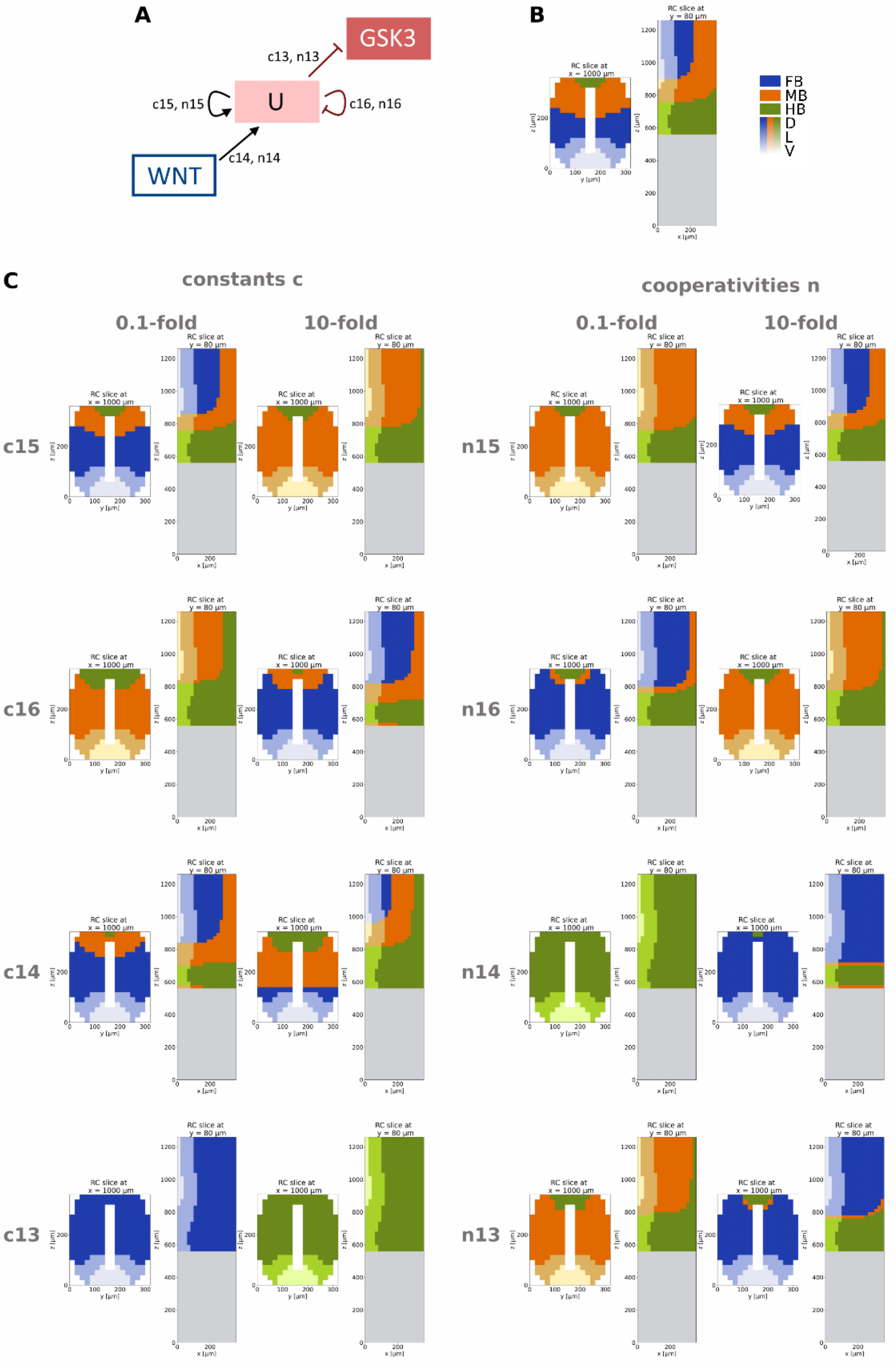

Figure S2: U-node parameter influence on patterning results. **A:** Illustration of the parameters that directly connected to the U-node levels; i.e. either targets or modulators. Shown are rate constants (c) and Hill coefficients (n). The numbers correspond to the parameters and equations in the Transparent Methods. **B:** Pattern resulting from the standard parameter set on the cylindrical tube (Supplementary Figure S3). **C:** Resulting patterns after scaling individual parameters 0.1-fold / 10-fold. Note that U self-activation /-repression (parameters 15, 16) exhibit converse sensitivity to parameter changes and primarily modulate the extent of the MB domain. Moreover, the pattern is less sensitive to the modulation of parameters associated with direct WNT than it is to the ones that act downstream repressing GSK3. This illustrates that the main function of U is to buffer the WNT signal for the rostrocaudal network branch.

Figure S3. Simplified model of the neural tube, Related to Figure 3

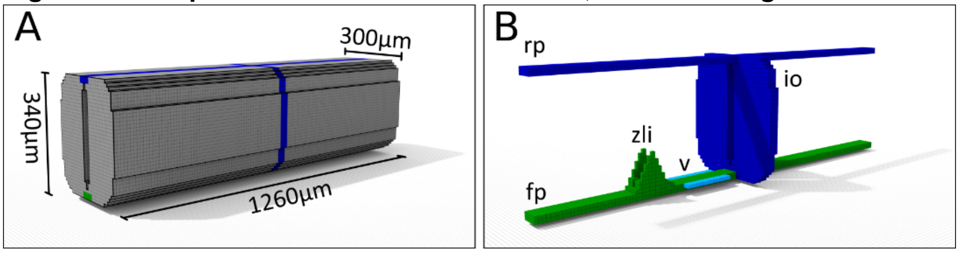

Figure S3: Simplification of neural tube geometry and important secretion points. **A:** Neural tube with secretion areas. **B:** Secretion areas including isthmic organizer (io), roof plate (rp), ventral midbrain (v), zona limitans (zli) and floor plate (fp).

Figure S4. Effect of tube geometry and secretion site setup on patterning, Related to Figure 3

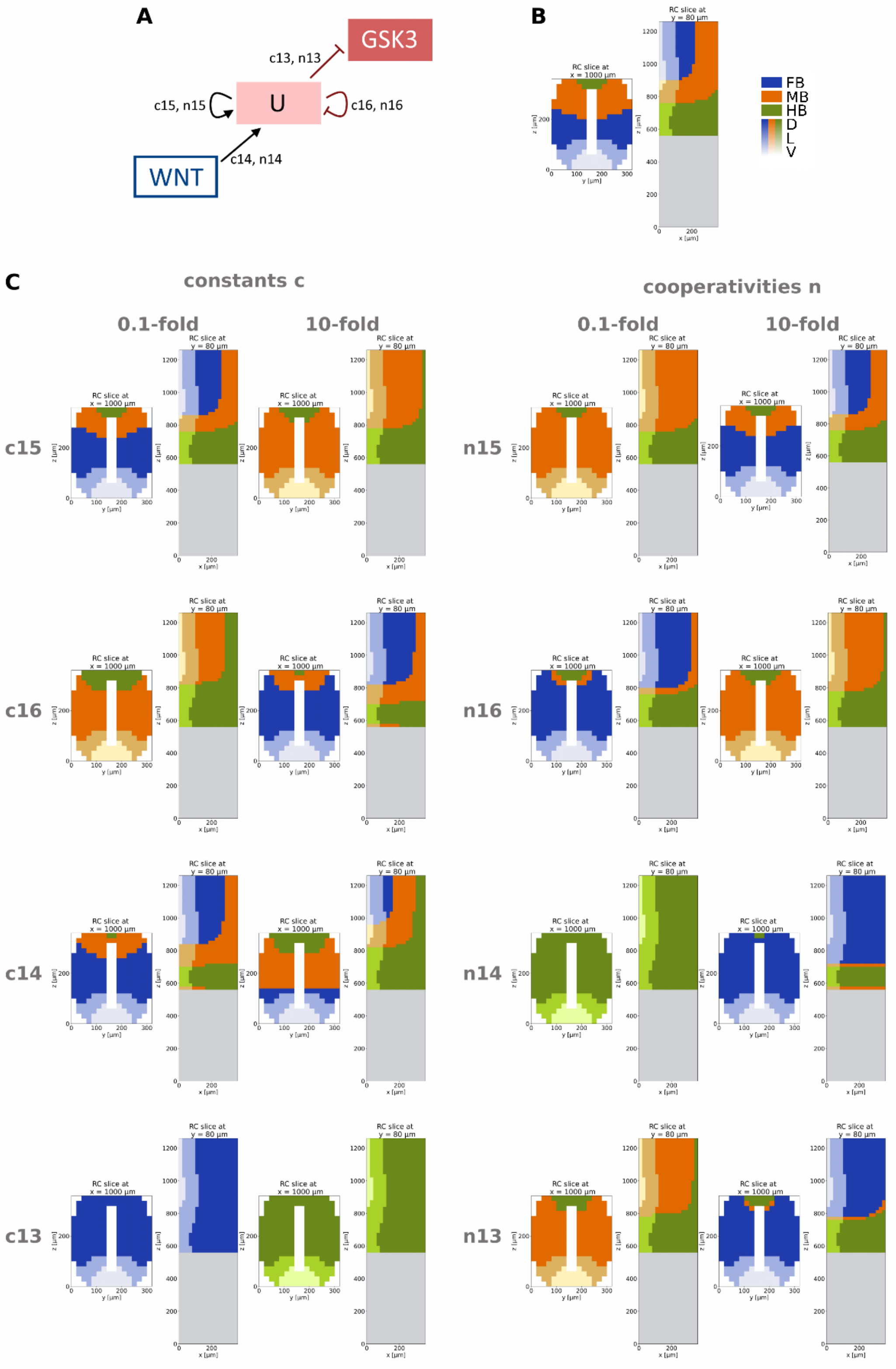

Figure S4: Effect of tube geometry and secretion site setup on patterning. The setup is indicated by the subtitle of each column (IO – isthmic organizer, ZLI – zona limitans, RP – roof plate, FP - floor plate). The top row shows patterning in the simplified tube model (Figure S2), while the bottom row displays the effect of the same setup for the realistic tube model (Figure 3b). Note that the roof plate (RP) signal strongly modulates the midbrain area and also causes stacking of the rostrocaudal brain.

Figure S5: SHH and WNT knockdown simulations, Related to Figure 5

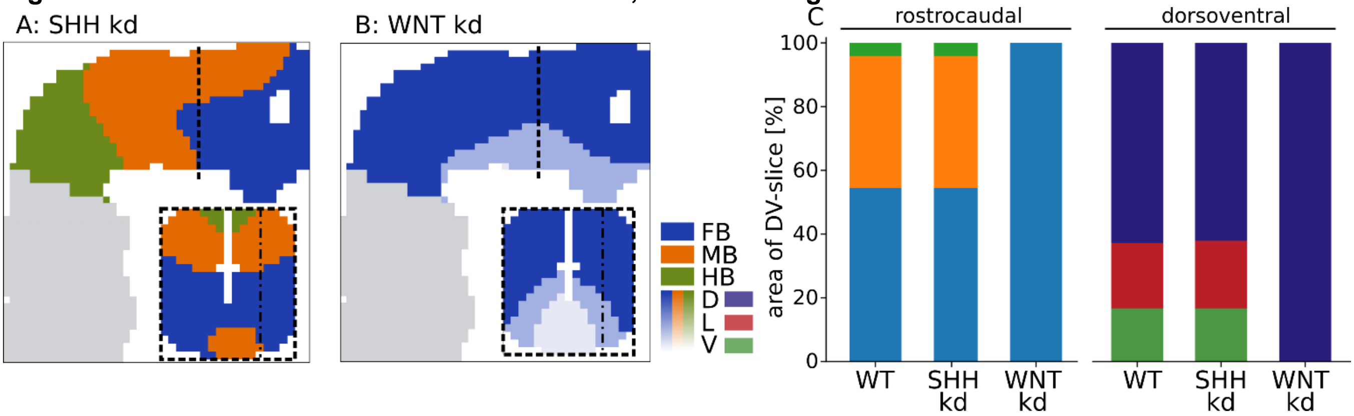

Figure S5: SHH and WNT knockdown simulations. **A:** Knockdown of SSH secretion. The rostrocaudal pattern was unaffected by the overexpression, whereas the dorsoventral pattern collapses and exhibits only D fate. **B:** Knockdown of WNT secretion. Predominantly, the rostrocaudal pattern was affected, which collapses and exhibits only FB fate. **C:** Quantification of the expression domain's sizes for the rostrocaudal and dorsoventral pattern analogous to Figure 4. Greyed areas are only shown for orientation and are not considered for pattern establishment. Dashed lines indicate the position of the transversal section (x = 580µm); dot-dashed lines indicate the position of the sagittal section (y = 80µm).
