# Supplementary material for: Neural tube patterning: from a minimal model for rostrocaudal patterning toward an integrated 3D model
Source: iScience. 2021 May 20;24(6):102559. doi: 10.1016/j.isci.2021.102559 (PMC8184516; doi:10.1016/j.isci.2021.102559)
Supplement: Document S2. supplemental information [file mmc2.pdf]

iScience

CellPress
OPEN ACCESS

Article

Neural tube patterning: from a minimal model for rostrocaudal patterning toward an integrated 3D model

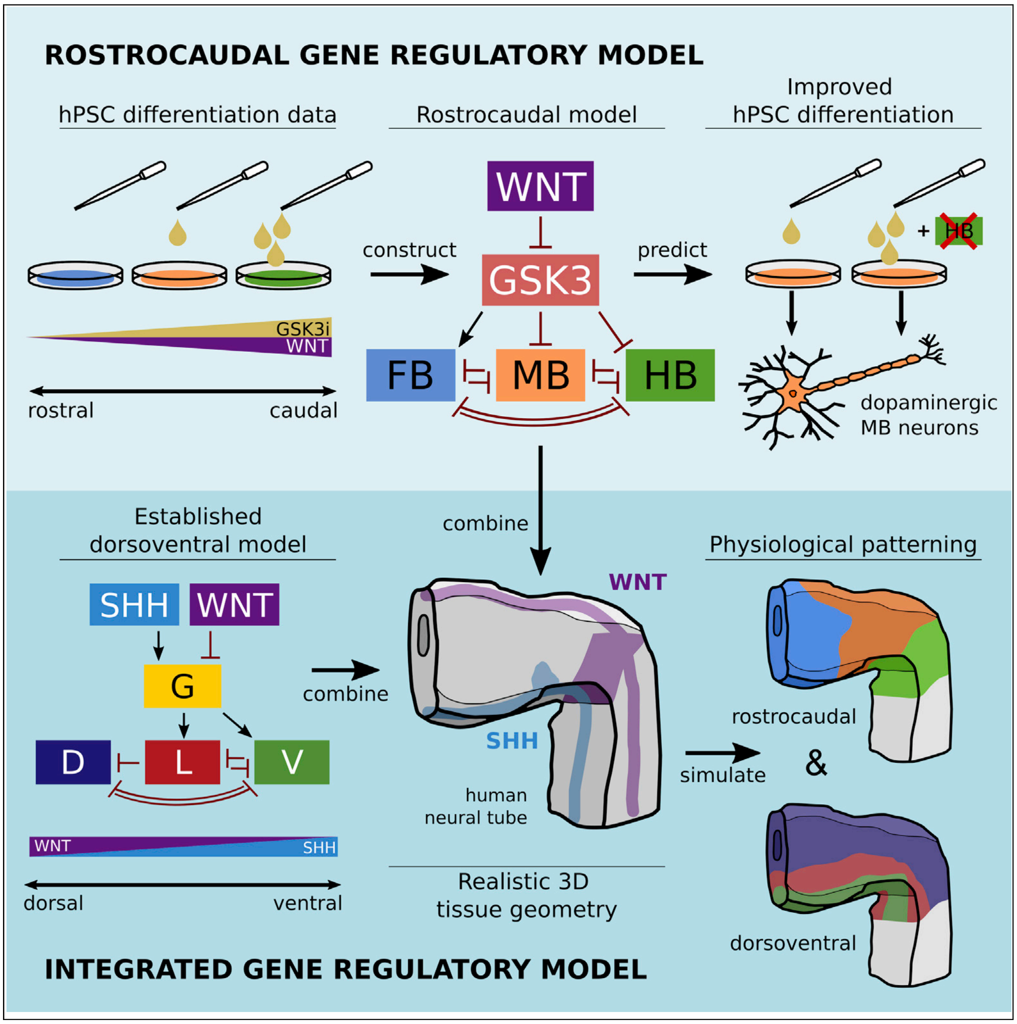

Max Brambach, Ariane Ernst, Sara Nolbrant, Janelle Drouin-Ouellet, Agnete Kirkeby, Malin Parmar, Victor Olariu

max.brambach@gmail.com (M.B.)
victor.olariu@thep.lu.se (V.O.)

Highlights

WNT regulates rostrocaudal neural tube patterning via a tristable network motif.

Blocking hindbrain fate may improve generation of dopaminergic neurons from hPSCs.

Combined rostrocaudal and dorsoventral model correctly patterns 3D neural tube.

Models of *in vivo* processes can be built modularly using data from diverse sources.

Brambach et al., iScience
■■, 102559
■■, 2021 © 2021 The Author(s).
https://doi.org/10.1016/j.isci.2021.102559

Please cite this article in press as: Brambach et al., Neural tube patterning: from a minimal model for rostrocaudal patterning toward an integrated 3D model, iScience (2021), https://doi.org/10.1016/j.isci.2021.102559

iScience

CellPress
OPEN ACCESS

Article

Neural tube patterning: from a minimal model for rostrocaudal patterning toward an integrated 3D model

Max Brambach,[1,*] Ariane Ernst,[1] Sara Nolbrant,[2] Janelle Drouin-Ouellet,[3] Agnete Kirkeby,[2] Malin Parmar,[2]
Q1 and Victor Olariu[1,4,*]

SUMMARY

Rostrocaudal patterning of the neural tube is a defining event in vertebrate brain development. This process is driven by morphogen gradients which specify the fate of neural progenitor cells, leading to the partitioning of the tube. Although this is extensively studied experimentally, an integrated view of the genetic circuitry is lacking. Here, we present a minimal gene regulatory model for rostrocaudal patterning, whose tristable topology was determined in a data-driven way. Using this model, we identified the repression of hindbrain fate as promising strategy for the improvement of current protocols for the generation of dopaminergic neurons. Furthermore, we combined our model with an established minimal model for dorsoventral patterning on a realistic 3D neural tube and found that key features of neural tube patterning could be recapitulated. Doing so, we demonstrate how data and models from different sources can be combined to simulate complex *in vivo* processes.

Q3 Q4 Q5 Q6 Q2

INTRODUCTION

In Parkinson's disease (PD), the dopaminergic neurons (DA) of the substantia nigra of the brain undergo pathological deterioration. The resulting deficiency in striatal dopamine leads to symptoms such as tremor, bradykinesia and rigidity that can currently not be relieved sufficiently by pharmacological treatment (Chinta and Andersen, 2005; Takagi et al., 2005). Today, transplantation therapy is the most promising approach to achieving PD recovery (Lehnen et al., 2017); several studies and clinical trials have demonstrated restoration of dopaminergic activity and partial symptom relief for up to 18 years post-transplantation (Barker et al., 2015; Kefalopoulou et al., 2014; Takagi et al., 2005). The conventional source of cells for these transplants, human fetal ventral mesencephalic tissue – which contains a high concentration of dopaminergic neuroblasts (Kefalopoulou et al., 2014; Lindvall, 2012) – is naturally scarce, ethically controversial and suffers from highly variable results (Lindvall, 2012). Recently, it has been suggested that DA progenitor generation by differentiation of human pluripotent stem cells (hPSCs) presents an alternative approach. Studies in animal models have shown that hPSC-derived DA progenitors are equivalent to fetal cells in terms of subtype specific marker expression, controlled dopamine release and functional PD symptom relief (Grealish et al., 2014; Kirkeby and Parmar, 2012a, 2012b; Kriks et al., 2011; Lehnen et al., 2017; Steinbeck et al., 2015). To date, various protocols have been developed to efficiently derive DA progenitors from human embryonic stem cells; many of them focusing on tuning a combination of WNT, SHH and FGF8 with great success (Adil et al., 2017; Cho et al., 2008; González et al., 2013; Kim et al., 2002; Kirkeby et al., 2012; Nolbrant et al., 2017). These protocols are inspired by the mechanisms and actors found in the development of the neural tube *in vivo*. Therefore, a better understanding of correct and reliable DA differentiation *in vivo* would not only shed light on the development of the early brain, but also contribute to improving current *in vitro* protocols for midbrain DA neuron generation.

In vivo neural patterning is a complicated and concerted process that relies on the three-dimensional diffusion of various morphogen signals in an uneven geometry. In humans, the current understanding of this process can be summarised as follows. By the end of the fourth week of embryonic development the progenitor of the embryo's brain, the neural tube, is patterned along the rostrocaudal axis into three distinct regions that develop into forebrain, midbrain and hindbrain (Gasser, 1975). In addition, patterning occurs along the dorsoventral axis, setting up numerous progenitor cell types such as motor neurons and

[1]Computational Biology and Biological Physics, Department of Astronomy and Theoretical Physics, Lund University, Lund, 223 63, Sweden

[2]Departments of Experimental Medical Science and Clinical Sciences, Wallenberg Neuroscience Center, and Lund Stem Cell Center, Lund University, 221 84 Lund, Sweden

[3]Faculté de Pharmacie, Université de Montréal, Montréal, QC, H3T 1J4, Canada

[4]Lead contact

*Correspondence: max.brambach@gmail.com (M.B.), victor.olariu@thep.lu.se (V.O.)

https://doi.org/10.1016/j.isci.2021.102559

This is an open access article under the CC BY license (http://creativecommons.org/licenses/by/4.0/).

interneurons (Wilson and Maden, 2005). The formation and subdivision of the neural tube is the result of extracellular morphogens that are released in specific areas of the tube, establishing concentration gradients. The cells of the neural tube are able to interpret the local concentration of these gradients and take fate decisions accordingly. Since gene regulatory networks have been shown to control pattern formation in multiple tissues (Briscoe, 2019; Peter et al., 2012; Peter and Davidson, 2015; Sagner and Briscoe, 2017), the developing vertebrate neural tube pattern might be the response of transcriptional circuitry inside neural progenitor cells to morphogen gradients. Dorsoventral patterning is mainly controlled by opposing signaling gradients of WNT/BMP from the roof plate, and SHH from floor plate cells (Ulloa and Martí, 2009) and the zona limitans intrathalamica (Carlson, 2013). Neural tube rostrocaudal patterning is mainly governed by WNT-signalling, emerging from the isthmic organizer (Tao and Zhang, 2016) and the midbrain floor plate in the cephalic flexure (Joksimovic et al., 2009; Prakash et al., 2006). It has been shown *in vitro* that controlling the gradient of the WNT-signalling, using Glycogen synthase kinase 3 (GSK3) inhibitors either in different cell cultures (Kirkeby et al., 2012) or in microfluidic devices (Rifes et al., 2020a; Rifes et al., 2020b), acting on differentiating human embryonic stem cells can lead to progressive caudalisation from forebrain to hindbrain. However, the gene regulatory network acting downstream of WNT-signalling that regulates this patterning processes has not been elucidated.

In this study, we propose a minimal gene regulatory network model for rostrocaudal neural tube patterning. The transcriptional circuit topology was determined in an unbiased way and the model parameters were optimised using gene expression data from a study on hPSCs, which were cultured in conditions with varied levels of WNT-signalling (Kirkeby et al., 2012). Subsequently, model parameter sensitivity analysis revealed the key interactions that drive the patterning process. Moreover, knockdown and overexpression model simulations gave insight into the regulation of the patterning and enabled the prediction of more efficient protocols for the generation of DA neurons *in vitro*. Furthermore, our optimised model was combined with the model controlling dorsoventral patterning proposed in (Balaskas et al., 2012) to simulate the pattern of the neural tube in a realistic, three-dimensional (3D) geometry, since recent advances in PD research incorporating organoids (Monzel et al., 2017; Smits et al., 2019) make it necessary to not only investigate morphogen and gene interactions but also the effects of tissue geometry on neural patterning. This way, we investigated which parts of the neural tube's pattern could be explained by our minimal 3D model. Moreover, by integrating theoretical models and quantitative data from different sources, we propose a 'divide and conquer' framework for the successive development of mathematical models capable to elucidate complex biological processes - for which often comprehensive datasets are not obtainable.

RESULTS

Gene expression of GSK3i treated cells clusters three-fold

As a first step toward a minimal gene regulatory network for rostrocaudal neural tube patterning, we aimed to identify the essential genes involved in this process. To this end, we used experimental data consisting of the expression levels of a selection of genes recorded in hPSCs for varying concentrations of the GSK3 inhibitor CHIR99021 (CT) obtained via quantitative PCR (qPCR). This dataset has been published in (Kirkeby et al., 2012). GSK3 is a negative regulator of canonical WNT-signalling, i.e. low GSK3 activity emulates strong WNT activity and vice versa. To identify the genes that correspond to the patterned brain regions, we determined the correlation between the gene expression across the varied CT concentrations. For this, we used a hierarchical agglomerative clustering approach, resulting in a correlation cluster map (Müllner, 2011) (Figure 1A). From this analysis, we identified the three major clusters with positive correlation. Examining the expression levels of these three gene clusters, three distinct regions of gene expression were found for varied levels of CT (Figure 1B). For the gene regulatory network model parameter and topology optimisation we selected one representative gene for each brain region. For the forebrain (FB) region we used forkhead box G1 (FoxG1), since it has been shown to be involved in telencephalon fate (Kobayashi et al., 2002; Toresson et al., 1998; Xuan et al., 1995). The midbrain (MB) region was represented by engrailed 1 (EN1), due to its key role in DA neuron specification (Alves dos Santos and Smidt, 2011; Kouwenhoven et al., 2017). For the hindbrain (HB) region we chose HOXA2, as it is reported to be a central regulator in hindbrain fate (Barrow et al., 2000; Davenne et al., 1999). The selection of one representative gene per brain region enabled us to construct a minimal gene regulatory network to capture the dynamics of neural tube patterning. However, it is important to note that this does not necessarily imply that the selected genes are interacting directly or are primarily regulating cell fate specification. Therefore, in the following results, the labels FB, MB and HB are used, when referring to the genes.

Please cite this article in press as: Brambach et al., Neural tube patterning: from a minimal model for rostrocaudal patterning toward an integrated 3D model, iScience (2021), https://doi.org/10.1016/j.isci.2021.102559

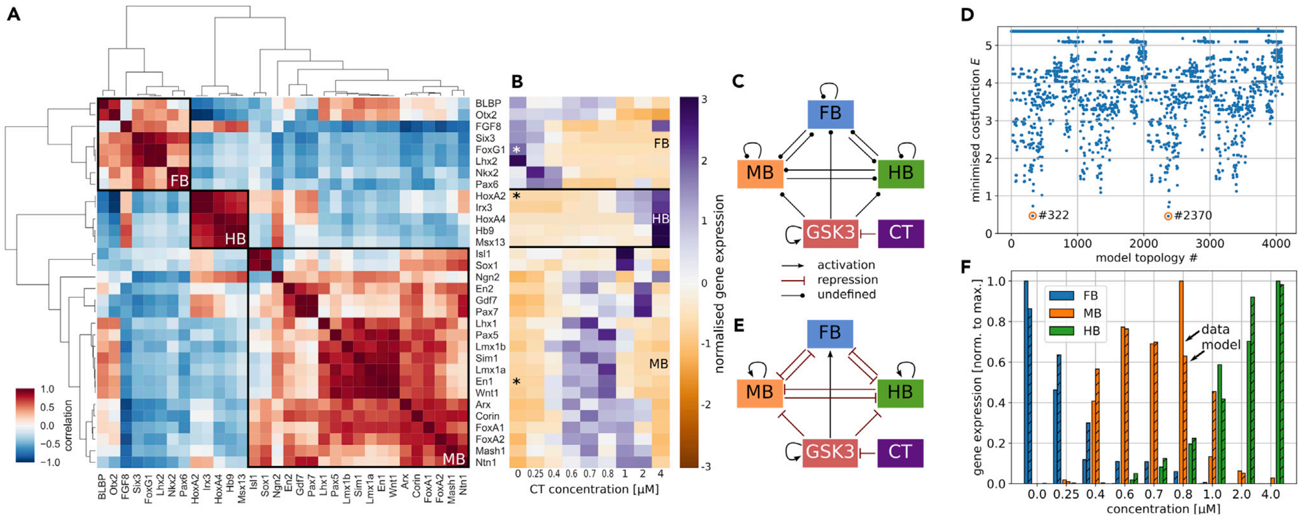

Figure 1. Experimental data from *in vitro* neural patterning experiments and model selection

(A) Correlation cluster map of the different brain region specific genes across CT concentrations. Three clusters were identified, which correspond to the three brain regions.

(B) Normalised gene expression of FB, MB and HB specific genes for different concentrations of the GSK3 inhibitor CT. Genes marked with asterisk are used to represent the brain regions in the following results.

(C) Model network with all possible interactions between the brain region nodes and their reaction dependency on **GSK3 activity. CT** controls GSK3, which is self-activating to allow for steady-state GSK3 activity. During the topology selection, all undefined interactions were either activation or repression.

(D) Minimal optimised cost function value for all possible network topologies. Two optimal topologies with similar and overall lowest cost functions were identified.

(E) Best model as determined by the topology selection. The three brain regions formed a tristable switch, controlled by the level of GSK3, which is in turn controlled by CT concentration. Note that GSK3, MB and HB are self-activating to allow for respective constant steady state gene expression. Degradation of GSK3, FB, MB, HB not illustrated. F: Gene expression levels of the three brain region specific genes given by the experimental data (plain bars) and the model (striped bars) for varied concentrations of CT.

Unbiased topology selection identifies a tristable switch configuration

With the key players of the network identified, the next step toward a minimal gene regulatory network was the determination of their interactions i.e. the connection between the nodes. These nodes are FB, MB, HB, GSK3 and CT (Figure 1C). We determined the interaction between these nodes and the resulting network topology in an unbiased way by optimising all possible configurations toward the experimental data. The only two interactions that were pre-defined are the inhibition of GSK3 by CT, which resulted in the necessity of GSK3 self-activation to allow for steady state activity of GSK3. The remaining interactions could be either activation or repression, which made for 4,096 possible network configurations. The configurations that yielded the lowest cost function E_d (see STAR Methods) after parameter optimisation were considered as the optimal configurations (Figure 1D); two topologies satisfied this criterion. These two configurations only differed in the self-interaction of FB, which was activation in one case and repression in the other. However, the corresponding rate constant c_0 (see STAR Methods) had a significantly smaller value compared to the other rate constants and therefore we concluded that, for the purpose of this minimal model, FB does not self-interact.

To verify that the found topology was not specific to the selected representative genes, we optimised our model toward all remaining 759 permutations of region-specific genes (Figure S1A) and found that in the majority of cases, the model was able to recapitulate the data. Moreover, we randomly selected 16 permutations of region-specific genes and repeated the topology selection as described before, (Figure S1B). This revealed that in more than 80% of the cases (13/16) the found optimal topology was very similar or identical to the initial one. These results demonstrate that the found tristable switch topology generally captures the interaction between the region-specific genes.

The model resulting from the unbiased topology selection is shown in Figure 1E. In this model, the three brain regions are mutually repressive and hence form a tristable switch. MB and HB fate are downregulated by GSK3, whereas FB fate is positively regulated. Also, FB does not self-interact and therefore directly

follows GSK3 activity when the latter is active at high levels. Furthermore, FB and MB are mutually repressive leading to a posterior limitation of the FB domain by the MB domain, recapitulating findings from *in vivo* studies, where an impairment of the MB domain led to a posterior expansion of the FB domain (Scholpp and Brand, 2003). For lower activity of GSK3, FB is no longer strongly expressed and can be fully repressed by the presence of MB or HB. This reduces the network to a co-repressive motif of MB and HB fate for lower GSK3 activity levels, which is controlled by their mutual repression strength and the repression through GSK3. In fact, the corresponding parameters were determined such that HB is repressing MB much stronger than vice-versa. GSK3's repression on HB is even stronger compared to its repression of MB. It follows that for low GSK3 activity levels HB will dominate over MB and vice versa for higher GSK3 levels. This dynamic recapitulates the interactions occurring when the MB-HB boundary is induced, with MB and HB specific genes (Otx2, Gbx2) repressing each other (Brafman and Willert, 2017; Wittman et al., 2009). Additionally, Fgf8 expression in the MB-HB boundary is induced by HB and repressed by MB while upregulating HB and downregulating MB, leading to a nett asymmetrical repression pattern where HB represses MB more strongly than vice versa (Brafman and Willert, 2017; Wittman et al., 2009). In our minimal model this interaction via Fgf8 is encoded implicitly in the asymmetrical interaction between MB and HB. Moreover, the reduction of the tristable switch motif to a bistable motif for lower GSK3 activity levels indicates that the MB-HB boundary-like interactions are a central element of neural tube patterning.

Figure 1F shows the brain regions' expression levels obtained from model simulations together with the experimental data used for the parameter optimisation. For most CT concentrations, model and data show high correlation. A significant deviation between model and data only occurred for the CT concentrations 0.8 μM and 1.0 μM. However, it is important to note that the overall structure of the brain regions' response to varied CT concentration in this minimal model is close to the experimental data.

Sensitivity analysis of model parameters reveals key interactions

Next, we examined the sensitivity of the model output to changes of the model parameters to identify the key interactions. *In vitro* data were used as reference and consequently the results are given in terms of the cost function E_d, which was defined as the mean squared distance between model and data. Figures 2A and 2B show that the most sensitive rate constants are c_4, c_8 and c_{12}, which correspond to the self-activation of MB, HB and GSK3. A similar sensitivity pattern was found in the Hill coefficients n_i, for which also n_4, n_8, and n_{12}were the most sensitive parameters, illustrating cooperativity and ultrasensitivity for MB, HB and GSK3. The most sensitive degradation rates were also the ones corresponding to the half-lives of MB, HB and GSK3 respectively (δ_2, δ_3 and δ_4). This implied that the most sensitive part of the model was the balance between self-activation and inhibition of MB, HB and GSK3 activity and the resulting steady state gene expression level. This was to be expected, since those expression levels are controlling which node of the tristable switch is active. FB does not self-interact, and its expression is therefore strongly correlated to the activity of GSK3 at high GSK3 activity levels. Also, high GSK3 activity levels lead to repression of MB and HB, whereas at low GSK3 activity levels MB or HB repress the expression of FB, explaining the low sensitivity of the model to changes of the FB degradation rate δ_1. The sensitivity of the repressions between FB and HB ($c_{2,10}, n_{2,10}$) was low, because either (at high GSK3 activity levels) HB was repressed by GSK3 or (at low GSK3 activity levels) FB was turned off. In both cases the sensitivities of the rate constants and Hill coefficients were low because the corresponding concentrations were close to zero. Another weak sensitivity was found in the repression of HB by MB (c_9, n_9), implying that the HB level was more dependent on its self-balance and repression by GSK3 than on repression by MB. Figure 2C shows the mean cost function change when all parameters were varied. We observed that the model was not sensitive to small variations of the model parameters. The mean cost function change for the variation of the whole parameter set of $\pm$ 10 % was similar to the maximum cost function change for the variation of an individual parameter, illustrating that the model is robust under the variation of parameters.

The sensitivity analysis showed that the key parameters of our model were the self-interactions of MB, HB and GSK3, since they not only controlled their own expression and activity level, but also which node of the tristable switch was active. Furthermore, the general dynamics of the model suggested that FB was proportional to GSK3, MB and HB were anti-proportional to GSK3, and the extent of the MB peak was controlled by the HB expression level. Following this reasoning, we formulated the hypothesis that knockdown of FB would not have a strong effect on the extend of the MB domain. However, knockdown of HB would remove the repression of MB for low GSK3 activity levels and would therefore lead to an extended MB region that includes the former HB region.

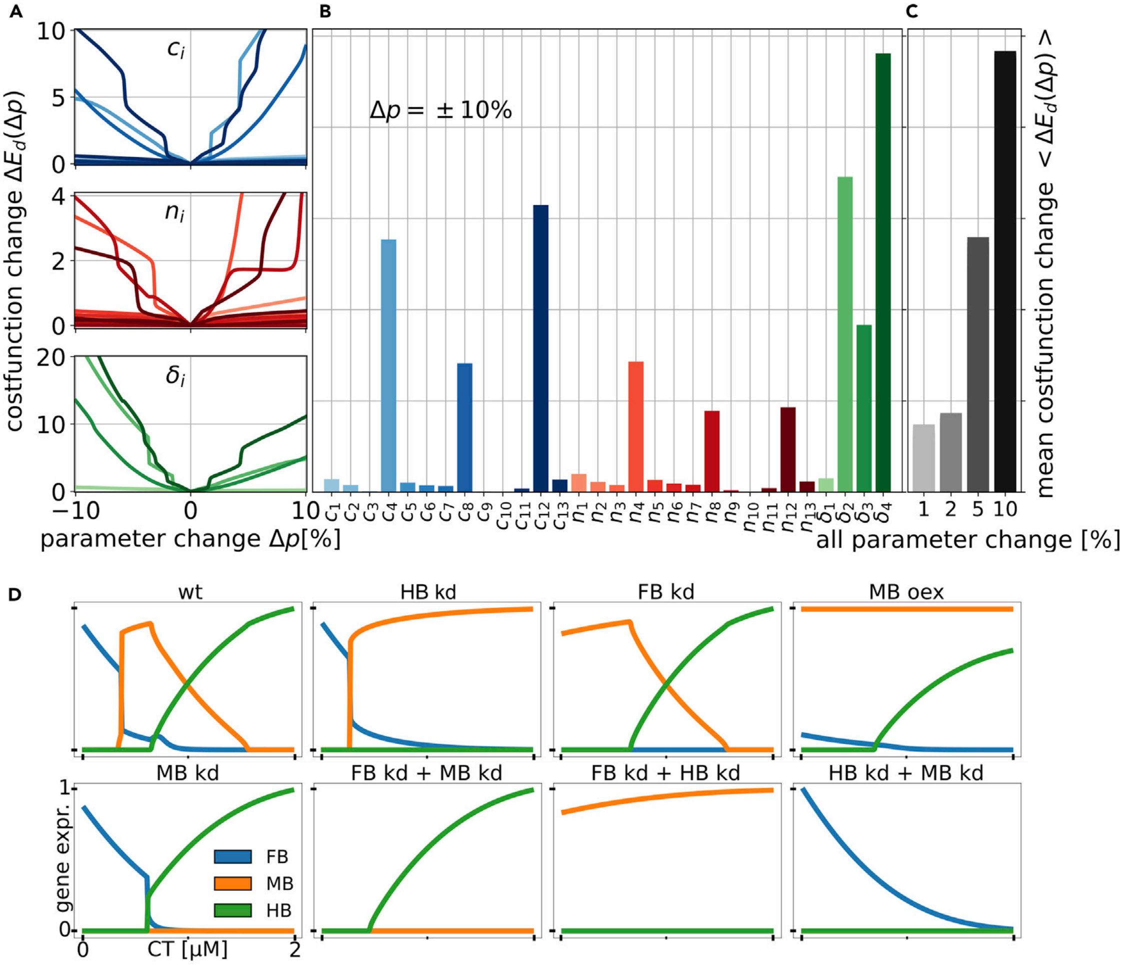

Figure 2. Sensitivity analysis of the model parameters and kd/oex predictions

(A) Fold-change of the cost function $\Delta E_d(\Delta p)$, with Δp being a parameter set with one parameter varied between 0 and 10% relative to the optimised parameter set.

(B) Mean cost function change $\langle \Delta E(\Delta p) \rangle$ across $\Delta p = \pm 10\%$ relative to the optimised value.

(C) $\langle \Delta E_d(\Delta p) \rangle$ for all parameters varied $\pm$ different relative amounts to the optimised parameter set.

(D) HB kd showed strong MB expression for CT > 0.2 µM. FB kd lead to strong MB expression for CT < 0.3 µM and wt behavior for higher CT concentrations. MB oex suppressed FB expression and lead to mixed MB/HB expression for higher CT concentrations. MB kd gave insight into the interaction between HB and FB and the double kd's highlight the reaction of the brain regions' gene expressions to the CT concentration. Axes are the same for all panels, gene expressions are scaled between 0 and 1.

Knockdown and overexpression simulations showed that only HB knockdown increases the MB domain

To test this hypothesis and to gain more insight into the transcriptional regulation of this patterning, we simulated knockdown (kd) and over-expression (oex) scenarios (Figure 2D). HB kd did not influence the interaction between FB and MB and lead to strong MB expression for CT > 0.2 µM, resulting in a more than five-fold larger CT window for MB fate. Knockdown of FB similarly did not influence the interaction between MB and HB and led to strong MB expression for CT < 0.3 µM. However, this only doubled the extent of the CT window for MB fate. Interestingly, when overexpressing MB, FB expression was lost, but the HB expression pattern was similar to wildtype. This gave rise to mixed MB-HB expression for CT > 0.4 µM and resulted in a CT window for HB fate similar to the FB kd case. Knockdown of MB revealed that FB and HB have the same mutually exclusive interaction as FB and MB in the wildtype case. This produced a slightly larger FB domain, whereas the HB domain was almost unaffected in extent or shape. The double knockdown simulations of FB and MB showed that HB expression requires a threshold CT concentration of CT > 0.2 µM and reacts positively to increasing CT past that threshold. The observed 'all off' state

Please cite this article in press as: Brambach et al., Neural tube patterning: from a minimal model for rostrocaudal patterning toward an integrated 3D model, iScience (2021), https://doi.org/10.1016/j.isci.2021.102559

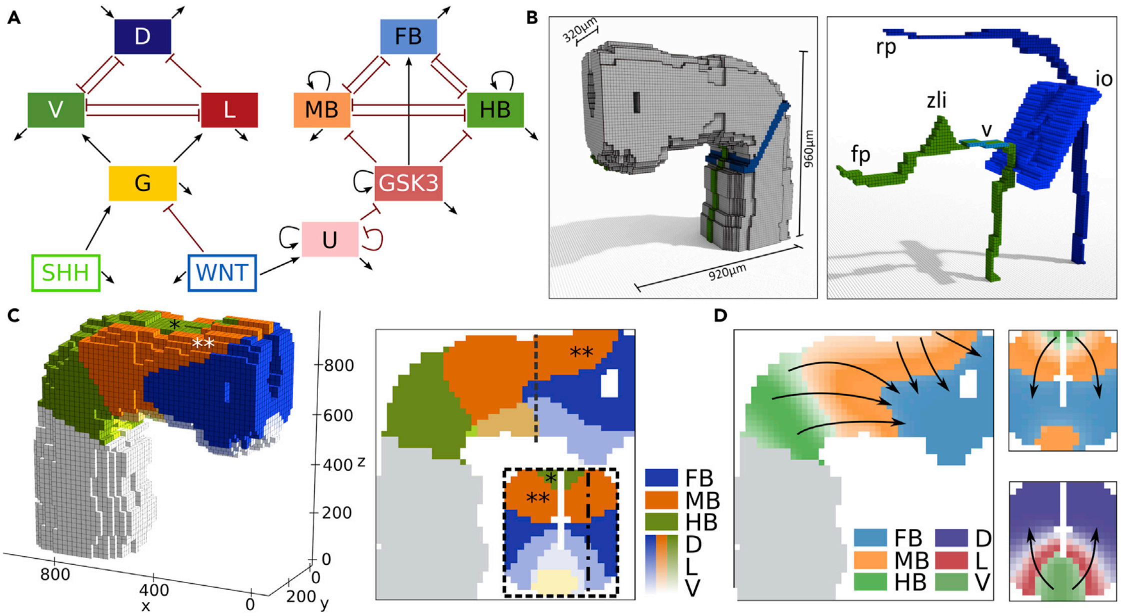

Figure 3. In silico patterning of the neural tube

(A) Complete model for neural tube patterning. Left branch: dorsoventral model by Balaskas et al., controlled by antagonistic action of SHH and WNT. G: Gli, D: dorsal (Pax6), L: Lateral (Olig2), V: ventral (Nkx2.2). Right branch: Proposed model for rostrocaudal patterning governed by WNT action.

(B) Three-dimensional model of neural tube geometry and the morphogen secretion sources. SHH (green): floor plate (fp) and zona limitans (zli); WNT (blue): roof plate (rp), the ventral midbrain (v) and isthmic organiser (io).

(C) Steady-state distribution of the patterned regions after applying the complete model. The geometry of the tube caused dorsoventral stacking of the rostrocaudal gene domains (* HB stacking, ** MB stacking).

(D) Rostrocaudal and dorsoventral steady-state pattern. Arrows indicate the direction of pattern establishment i.e. the main direction of morphogen action. Greyed areas in C and D are only shown for orientation and are not considered for pattern establishment. Dashed line indicates the position of the transversal section (x = 580μm); dot-dashed line indicates the position of the sagittal section (y = 80μm).

found for low CT concentrations corresponds to a state that is not captured by our minimal model and we speculate that this state may be analogous to the multi-lineage priming state observed in other systems (Zhou et al., 2019). Knockdown of MB and HB illustrated that FB expression is negatively regulated by CT. Interestingly, the knockdown of FB and HB showed that MB reacts only weakly to CT at low levels. This implies that MB is only weakly regulated by CT itself. These results confirmed the findings from the parameter sensitivity analysis and suggest that protocols for the generation of DA MB neurons could be made more robust and independent of precise CT concentration by repressing HB fate. This would significantly increase the window of CT concentration that results in MB fate, while the repression of FB fate or the promotion of MB fate via overexpression would not lead to the same result.

Application of model to realistic geometry revealed dorsoventral stacking of rostrocaudal pattern

To further test the potential of the minimal rostrocaudal model, we merged it with the model of Balaskas et al. for dorsoventral patterning (Balaskas et al., 2012) and used the combined model to simulate the steady state pattern of the human neural tube *in vivo*. The combined model is shown in Figure 3 A. WNT signaling controls both dorsoventral and rostrocaudal patterning and therefore, the two model branches are linked through the WNT node. In the rostrocaudal network branch, a buffer node (U) translates the WNT signal into the GSK3-inhibition as achieved by CT in the *in vitro* case. On the dorsoventral network branch, Gli expression is repressed by WNT signaling and activated by SHH activity (Ulloa and Martí, 2009). Gli expression acts as selector for dorsoventral fate – like GSK3 in the rostrocaudal case – by activating the expression of ventral fate (Nkx2.2, V) and lateral fate (Olig2, L), which are mutually repressive. Both V and L repress the expression of dorsal fate (Pax6, D), resulting in a reduced tristable switch network motif.

We further investigated the role of the introduced U-node by analysing the sensitivity of the resulting pattern to changes of its associated parameters (Figure S2) and found that U self-activation and -repression exhibit

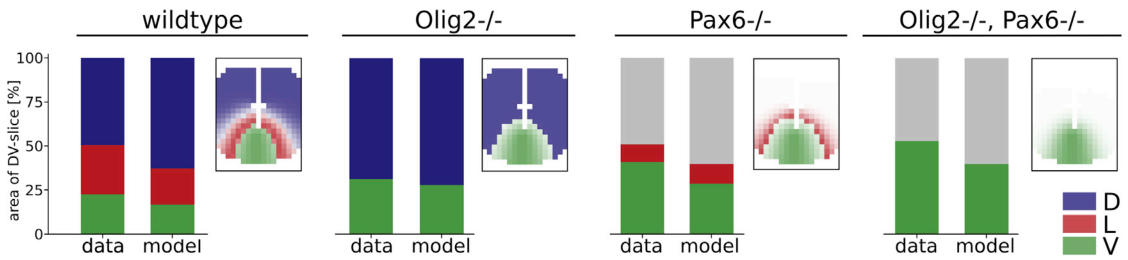

Figure 4. Comparison of the simulated pattern with data from wildtype and mutant mice

Data from (Balaskas et al., 2012); quantified model output is the percentage of the expressing area in the dorsoventral slice at x = 580μm (shown next to corresponding bar plot). Expression classification of the model was achieved via thresholding (see STAR Methods). Gray bars correspond to areas that exhibit no expression of either D, L, V above the set threshold.

converse sensitivity to parameter changes effectively controlling the extent of the MB domain. Moreover, the pattern was more robust to changes of parameters associated with the modulation of U by WNT than to changes of parameters associated with the modulation of GSK3 by U, indicating that indeed the main function of U is buffering. A biological interpretation of this WNT-U-GSK3 cascade could be the dynamics of the β-catenin destruction complex (Stamos and Weis, 2013). In this scenario, U would correspond to the activity of the Frizzled receptor which sequesters the destruction complex and effectively reduces the rate of β-catenin degradation, which in our model is represented inversely by the GSK3 activity level.

To explore effects of the curved tube geometry and morphogen secretion sites on patterning, we set up a spatial simulation of the network on a realistic three-dimensional model of the rostral neural tube (de Bakker et al., 2016) (Figure 3B). The WNT signal originates from the roof plate (rp), the ventral midbrain (v) and the isthmic organiser (io) while SHH is secreted from the floor plate (fp) and the zona limitans intrathalamic (zli) (Carlson, 2013; Joksimovic et al., 2009; Prakash et al., 2006). The diffusion dynamics of both morphogens were estimated based on their respective protein structure (see STAR Methods).

The calculated steady state pattern (Figure 3C) agreed with previous studies for the dorsoventral branch (Balaskas et al., 2012) and also yielded the anticipated rostrocaudal pattern in the ventral half of the neural tube. Interestingly, in the dorsal half of the neural tube, the additional WNT signal from the roof plate lead to a stacking of the rostrocaudal brain regions in dorsoventral direction (* HB stacking, ** MB stacking in Figures 3C and 3D), which resulted in an L shaped MB and HB region in a sagittal cross section. Repeated simulations with different tube geometries and varied secretion location setups revealed that the roof plate signaling is vital to increase the extent of the midbrain region, especially in the bent tube geometry, and that this is mainly responsible for the observed stacking (Figures S3 and S4).

A frontal cross section revealed that, caudally, the stacked rostrocaudal pattern did not overlap with the dorsoventral pattern. The former was contained in the Pax6 regime, whereas the latter fell fully inside the FB region. This resulted in five distinct regions in dorsoventral direction and nine in total.

To verify our patterning results, we compared the expression pattern of dorsoventral markers with experimental data from (Balaskas et al., 2012), which consists of WT, Olig2−/−, Pax6−/− and (Olig2−/−, Pax6−/−) expression patterns from mouse embryos. We simulated the WT and the mutant conditions and calculated the relative extent of the expression domains in specific slices of our model via thresholding. Generally, we found that our model predictions and the data agreed well with an average absolute deviation of 8 $\pm$ 4% (Figure 4). A systematic trend across most conditions was an under-prediction of the V domain which was on average 8 $\pm$ 3% too small. Since this behavior was present even in the absence of D (Pax6−/−), it could be that either the SHH secretion rate or the rate of Gli induced V activation in our model was underestimated or that this mismatch is rooted in the physiological difference between developing mouse embryos and humans.

Overexpression simulations of morphogen secretions show high sensitivity of neural progenitor cell fate to morphogen levels

To investigate the influence of morphogen secretion levels, we simulated overexpression of SHH and WNT production. SSH oex did not affect the rostrocaudal pattern, but significantly changed the dorsoventral pattern (Figure 5A). The V domain was drastically dorsally enlarged compared to the wildtype (WT) and

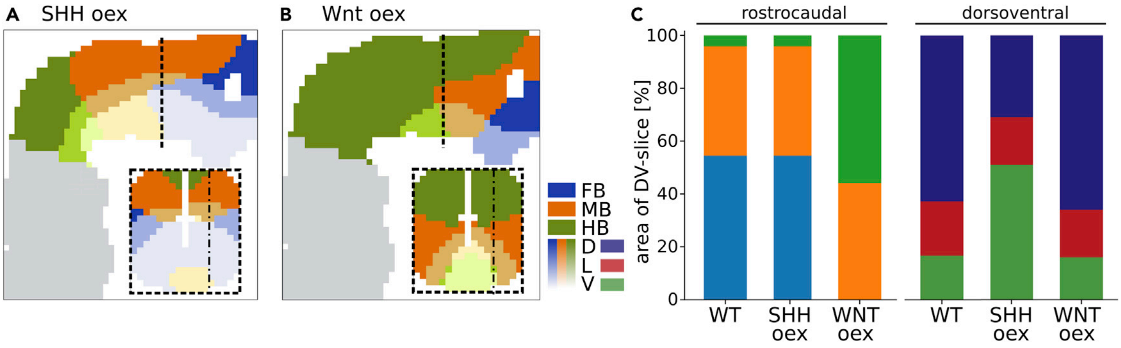

Figure 5. SHH and WNT overexpression simulations

(A) Overexpression of SSH secretion. The rostrocaudal pattern was unaffected by the overexpression, whereas the V domain was significantly larger than in the WT and the D domain was consequently smaller.

(B) Overexpression of WNT secretion. Predominantly, the rostrocaudal pattern was affected. The HB region was drastically enlarged, which led to smaller MB and FB regions. Moreover, the L-shape of WT HB and MB domains was lost.

(C) Quantification of the expression domain's sizes for the rostrocaudal and dorsoventral pattern analogous to Figure 4. Greyed areas are only shown for orientation and are not considered for pattern establishment. Dashed lines indicate the position of the transversal section (x = 580μm); dot-dashed lines indicate the position of the sagittal section (y = 80μm).

took up the whole ventral half of the neural tube. This led to a dorsal shift of the L domain, which, did not change in size. Consequently, the extent of the D domain was smaller. Through the dorsal shift of the pattern, the split of the dorsoventral domains occurred closer to the isthmic organiser and hence the extents of MB/HB + V/L domains were enlarged, leading to a decrease in size of the FB + L/V domains.

Overexpression of WNT had a small effect on dorsoventral patterning, decreasing the size of the V domain and shifting the L and D domains ventrally. However, the effects of this overexpression on the rostrocaudal pattern were more striking (Figure 5B). Most dominantly, the HB region was drastically enlarged, covering the whole caudal half of the neural tube and extending almost to the WT MB/FB boundary. This led to a ventral and caudal shift of the MB domain, which changed its characteristic L shape to a diagonal band and decreased its volume. Interestingly, through the dorsoventral stacking, the MB domain extended up to the caudal boundary of the neural tube. The FB domain was positioned in the ventral and rostral tip of the neural tube, covering less than half the volume of the WT FB domain. Combined, this led to a more pronounced dorsoventral stacking of the rostrocaudal brain regions in the caudal half of the neural tube, which gave rise to a large MB + L domain, while the volume of the WT HB + V domain was drastically reduced. Using the same quantification approach as for the comparison of the model with data from mouse embryos (Figure 5C), we found that the rostrocaudal pattern remained unchanged upon SHH oex. However, in the WNT oex condition the FB domain was absent and the HB domain was increased 11-fold. The MB domain was only enlarged by 3%, suggesting that the WNT expression level's main effect on the MB domain is positional. The dorsoventral pattern shows a converse response to the two overexpression conditions, with the pattern being almost unchanged upon WNT oex. SHH oex leads to a three-fold enlargement of the V domain which reduces the D domain to half its normal size. Interestingly, we found that, analogous to the rostrocaudal pattern, the lateral domain's size was unaffected by the overexpression (+1%).

Additionally, we simulated analogous knockdown (kd) conditions (Figure S5). We found that SHH kd does not affect the rostrocaudal pattern but abolishes the dorsoventral pattern, reducing it to only D expression and conversely, that WNT kd does not affect the dorsoventral pattern but reduces the rostrocaudal fate to only FB. This unsurprisingly illustrates the requirement of the morphogens for the establishment of the patterns.

DISCUSSION

We presented a simple mathematical model of rostrocaudal neural tube patterning. We determined its topology in an unbiased and data-driven fashion. The resulting configuration was that of a tristable switch of mutually repressing brain region fate under the control of the WNT target GSK3. Moreover, we showed that this configuration is not specific to a set of representative genes but is rather a general motif that was found

for most region-specific genes, indicating a central role for this network motif in rostrocaudal patterning. The unbiasedly discovered gene regulatory network has at its core genetic toggle switches consisting of cross-repressing transcription factors, which are network motifs that were shown to control patterning at tissue levels (Davidson, 2010). The toggle switch motif has the capability of translating the continuous signal of a morphogen gradient into an on-off behavior, in our case WNT gradient signal into e.g. FB off/MB on. Our multiple toggle switch model was able to recapitulate data from *in vitro* studies on hPSCs cultured at different WNT signaling levels (Kirkeby and Parmar, 2012a, 2012b) and exhibited similar dynamics as recent experiments done on a synthetic *in vitro* setup (Rifes et al., 2020a; Rifes et al., 2020b). Since, the toggle switch motif can control the boundaries of gene expression domains (Balaskas et al., 2012; Briscoe et al., 2000), parameter perturbation or manipulation (e.g. via knockdown, overexpression) of one of the factors can lead to changes in the resulting pattern. For our network model parameter sensitivity analysis and *in silico* overexpression and knockdown experiments suggested that the repression of HB fate, rather than the repression of FB fate or the promotion of MB fate, is the most promising strategy to increase efficiency and robustness of DA MB generation protocols. Repression of HB increased the window of supplied GSK3 inhibitor CT that resulted in MB fate. It will be interesting to see if this result can be validated experimentally; one option could be to use synthetic neural tube patterning similar to the work recently published in (Rifes et al., 2020a; Rifes et al., 2020b).

To obtain an integrated model for the pattern of the neural tube, we used a ‘divide and conquer’ approach, combining models and data relating to different sub-processes of the full patterning. One major difficulty in the development of theoretical gene regulatory models of *in vivo* processes is a lack of suitable, quantitative data for parameter estimation and model validation. Here, we aimed to demonstrate that this difficulty can be overcome by adhering to the following strategy: (1) Division of the process of interest into distinct subprocesses. (2) Development of minimal models for each subprocess. (3) Re-integration of the subprocesses in a physiological context. Following this approach, we show that it can be sufficient to rely on quantitative data from *in vitro* experiments which, is more readily available, easier to obtain and ethically less controversial.

By combining our rostrocaudal model with a previously published model for dorsoventral neural tube patterning we obtained an integrated model for the pattern of the neural tube controlled by the morphogens SHH and WNT. On the rostrocaudal side of this model, a buffer node U, which translates WNT signal to GSK3 inhibition, needed to be introduced to achieve stable steady state rostrocaudal patterning. This node combined with the GSK3 node possibly corresponds to the dynamics of the β-catenin destruction complex as part of the WNT signaling cascade and the need for it is an example for the dynamic difference between signaling cascades and direct modulation of gene expression. It should be noted that the configuration of the rostrocaudal branch of the integrated network is very similar to the dorsoventral branch, with both branches having a morphogen level-regulated multiple toggle switch configuration at their core. This is very much in line with the idea that network motifs like feedback and mutual repression predominate parts of the modular structure of gene regulatory networks (Alon, 2007; Davidson, 2010). To test the capabilities of the combined model, we simulated the patterning of the early human neural tube. For this, we set up a realistic three-dimensional model of the neural tube, which included the major morphogen secretion sites. The diffusion of the morphogens from these sites led to stable patterning of the tube into distinct regions, which are similar to existing data. However, we found that, in our model, the isthmic organiser (the posterior WNT secretion site) and the MB-HB boundary did not co-localise, with the latter being shifted anteriorly. This mismatch is most likely due to our model lacking of dynamic, brain region specific, auto-regulation of the WNT response during the induction of the MB-HB boundary as indicated in (Brafman and Willert, 2017). The integration of this sub-process into our model represents a promising next step for the expansion of the model. Interestingly, the bent geometry of the neural tube, combined with WNT secretion from the roof plate led to dorsoventral stacking of the rostrocaudal domains in the rostral half of the tube. Repeated simulations with different tube geometries and varied presences of the morphogen sources showed that roof plate signaling is mainly regulating the extent of the midbrain region and the dorsoventral stacking phenomenon. Unfortunately, no data was available to validate these model predictions. It would therefore be very interesting to find out if this is a shortcoming of the model or indeed a patterning mechanism. Data for such validation could be obtained for example by live microscopy of the neural tube in zebrafish embryos with fluorescently labeled region-specific genes. Lastly, we simulated the overexpression of morphogen secretion, which illustrated that cell fate and consequently neural tube patterning is sensitive to the abundance of morphogens seen by individual cells. In the WNT overexpression case, the model predicted an increase in HB fate, which is consistent with several previous studies (Itoh

and Sokol, 1997; McGrew et al., 1995; Pöpperl et al., 1997). SHH overexpression resulted in a drastic enlargement of the V-domain, shifting of the C-domain and size reduction of the D-domain, which is consistent with findings in (Jeong, 2004). Moreover, we find that the general sensitivity of neural progenitor cells to the abundance of WNT and SHH ligands predicted by the model to be in line with a recent study on hPSCs (Strano et al., 2020), implying that the secretion and distribution of morphogen ligands in a developing embryo have to be precisely and robustly regulated.

In summary, our 3D computational model demonstrates how the combination of sub-process models can be used to modularly construct a modeling framework leading to better understanding of complex and inaccessible biological processes. Moreover, the presented model for rostrocaudal neural tube patterning can, despite its simplicity, recapitulate the main features of neural tube patterning and neural differentiation in dependence of WNT signaling and predict strategies of improving the efficiency of DA MB generation protocols. In the future, targeted *in vitro* experiments are required to test the predictions of this model. Therefore, this study is only a first step toward an integrated and conclusive view for the 3D patterning of the neural tube *in vivo*.

Limitations of the study

It is important to note that the identification of gene expression clusters via hierarchical agglomeration is a correlative method. This means that the three identified clusters do not necessarily have to be connected mechanistically. Specifically, we can make no statement about whether the selected representative genes are interacting directly. Moreover, we noticed discrepancies between our minimal model and the used qPCR data for some concentrations of CT. This is most likely due to the simplicity of our model. However, the general dynamic of our model fits well with the data. Furthermore, the results of the integrated three-dimensional model could not be rigorously validated due to a lack of available data. Moreover, neural tube patterning is a dynamic process, yet our minimal 3D model only recapitulates an already patterned state in a static fashion. This means that cells can only receive signals from pre-defined secretion sites instead of being able to dynamically feedback and modulate the strength of morphogen secretion. Therefore, the model does not account for the dynamic induction of morphogen expression sites throughout development, such as the establishment of the MB/HB boundary.

STAR★METHODS

Detailed methods are provided in the online version of this paper and include the following:

- KEY RESOURCES TABLE
- RESOURCE AVAILABILITY
  - Lead contact
  - Materials availability
  - Data and code availability
- METHOD DETAILS
  - Clustering of gene expression data
  - Optimisation of model towards *in vitro* data
  - Similarity of model topologies
  - Differential equations
  - Model parameters and initial conditions:
  - Kd and oex implementation
  - Morphogen diffusivity
  - Quantification of gene expression
  - Computational methods
- QUANTIFICATION AND STATISTICAL ANALYSIS

SUPPLEMENTAL INFORMATION

Supplemental information can be found online at https://doi.org/10.1016/j.isci.2021.102559.

ACKNOWLEDGMENTS

The authors would like to thank Julia Deichmann for discussions at various stages of the project and for critical input on the manuscript. The authors would like to thank Patrik Eden, Anders Irbäck and Carsten

Peterson for valuable feedback on the project. VO gratefully acknowledges the support of the US National
Q9 Institutes of Health (USPHS grant R01HL119102) and Crafoordska Stiftelsen.

AUTHOR CONTRIBUTIONS

MB, AE, VO designed the study. MB, AE, VO wrote the manuscript. MB built the rostro-caudal model, conducted computational analysis. AE built the 3D computational model. MP, AK, JD-O, SN provided the experimental data. JD-O, SN explicated and conceptualised the use of experimental data. All authors provided inputs and comments on the manuscript.

DECLARATION OF INTERESTS

The authors declare no competing interests.

Received: December 9, 2020
Revised: April 16, 2021
Accepted: May 14, 2021
Published: ■■ ■■, ■■

REFERENCES

Adil, M.M., Rodrigues, G.M.C., Kulkarni, R.U., Rao, A.T., Chernavsky, N.E., Miller, E.W., and Schaffer, D.V. (2017). Efficient generation of hPSC-derived midbrain dopaminergic neurons in a fully defined, scalable, 3D biomaterial platform. Sci. Rep. *7*, 40573.

Alon, U. (2007). An Introduction to Systems Biology: Design Principles of Biological Circuits, Star (Chapman & Hall/CRC).

Alves dos Santos, M.T.M., and Smidt, M.P. (2011). En1 and Wnt signaling in midbrain dopaminergic neuronal development. Neural Dev. *6*, 23.

Balaskas, N., Ribeiro, A., Panovska, J., Dessaud, E., Sasai, N., Page, K.M., Briscoe, J., and Ribes, V. (2012). Gene regulatory logic for reading the sonic hedgehog signaling gradient in the vertebrate neural tube. Cell *148*, 273–284.

Barker, R.A., Drouin-Ouellet, J., and Parmar, M. (2015). Cell-based therapies for Parkinson disease-past insights and future potential. Nat. Rev. Neurol.

Barrow, J.R., Stadler, H.S., and Capecchi, M.R. (2000). Roles of Hoxa1 and Hoxa2 in patterning the early hindbrain of the mouse. Development *127*, 933–944.

Brafman, D., and Willert, K. (2017). Wnt/β-catenin signaling during early vertebrate neural development. Dev. Neurobiol. *77*, 1239–1259.

Briscoe, J. (2019). Understanding pattern formation in embryos: experiment, theory, and simulation. J. Comput. Biol. *26*, 696–702.

Briscoe, J., Pierani, A., Jessell, T.M., and Ericson, J. (2000). A homeodomain protein code specifies progenitor cell identity and neuronal fate in the ventral neural tube. Cell *101*, 435–445.

Byrd, R.H., Lu, P., Nocedal, J., and Zhu, C. (1995). A limited memory algorithm for bound constrained optimization. SIAM J. Sci. Comput. *16*, 1190–1208.

Carlson, B.M. (2013). Human Embryology and Developmental Biology, 5th ed. (Elsevier Inc).

Chinta, S.J., and Andersen, J.K. (2005). Dopaminergic neurons. Int. J. Biochem. Cell Biol. *37*, 942–946.

Cho, M.-S., Hwang, D.-Y., and Kim, D.-W. (2008). Efficient derivation of functional dopaminergic neurons from human embryonic stem cells on a large scale. Nat. Protoc. *3*, 1888–1894.

Davenne, M., Maconochie, M.K., Neun, R., Pattyn, A., Chambon, P., Krumlauf, R., and Rijli, F.M. (1999). Hoxa2 and Hoxb2 control dorsoventral patterns of neuronal development in the rostral hindbrain. Neuron *22*, 677–691.

Davidson, E.H. (2010). Emerging properties of animal gene regulatory networks. Nature *468*, 911–920.

de Bakker, B.S., de Jong, K.H., Hagoort, J., de Bree, K., Besselink, C.T., de Kanter, F.E.C., Veldhuis, T., Bais, B., Schildmeijer, R., Ruijter, J.M., et al. (2016). An interactive three-dimensional digital atlas and quantitative database of human development. Science *354*, aag0053.

Gasser, R.F. (1975). Atlas of Human Embryos (Harper & Row).

González, H., Contreras, F., Prado, C., Elgueta, D., Franz, D., Bernales, S., and Pacheco, R. (2013). Dopamine receptor D3 expressed on CD4 + T cells favors neurodegeneration of dopaminergic neurons during Parkinson's disease. J. Immunol. *190*, 5048–5056.

Grealish, S., Diguet, E., Kirkeby, A., Mattsson, B., Heuer, A., Bramoulle, Y., Van Camp, N., Perrier, A.L., Hantraye, P., Björklund, A., and Parmar, M. (2014). Human ESC-derived dopamine neurons show similar preclinical efficacy and potency to fetal neurons when grafted in a rat model of Parkinson's disease. Cell Stem Cell *15*, 653–665.

Harris, C.R., Millman, K.J., van der Walt, S.J., Gommers, R., Virtanen, P., Cournapeau, D., Wieser, E., Taylor, J., Berg, S., Smith, N.J., et al. (2020). Array programming with NumPy. Nature.

He, L., and Niemeyer, B. (2003). A novel correlation for protein diffusion coefficients based on molecular weight and radius of gyration. Biotechnol. Prog. *19*, 544–548.

Hong, L., and Lei, J. (2009). Scaling law for the radius of gyration of proteins and its dependence on hydrophobicity. J. Polym. Sci. Part B Polym. Phys. *47*, 207–214.

Hunter, J.D. (2007). Matplotlib: a 2D graphics environment. Comput. Sci. Eng. *9*, 90–95.

Itoh, K., and Sokol, S.Y. (1997). Graded amounts of Xenopus dishevelled specify discrete anteroposterior cell fates in prospective ectoderm. Mech. Dev. *61*, 113–125.

Jeong, J. (2004). Growth and pattern of the mammalian neural tube are governed by partially overlapping feedback activities of the hedgehog antagonists patched 1 and Hhip1. Development *132*, 143–154.

Joksimovic, M., Yun, B.A., Kittappa, R., Anderegg, A.M., Chang, W.W., Taketo, M.M., McKay, R.D.G., and Awatramani, R.B. (2009). Wnt antagonism of Shh facilitates midbrain floor plate neurogenesis. Nat. Neurosci. *12*, 125–131.

Kefalopoulou, Z., Politis, M., Piccini, P., Mencacci, N., Bhatia, K., Jahanshahi, M., Widner, H., Rehncrona, S., Brundin, P., Björklund, A., et al. (2014). Long-term clinical outcome of fetal cell transplantation for Parkinson disease. JAMA Neurol. *71*, 83.

Kim, J.-H., Auerbach, J.M., Rodríguez-Gómez, J.A., Velasco, I., Gavin, D., Lumelsky, N., Lee, S.-H., Nguyen, J., Sánchez-Pernaute, R., Bankiewicz, K., and McKay, R. (2002). Dopamine neurons derived from embryonic stem cells function in an animal model of Parkinson's disease. Nature *418*, 50–56.

Kirkeby, A., Grealish, S., Wolf, D.A., Nelander, J., Wood, J., Lundblad, M., Lindvall, O., and Parmar, M. (2012). Generation of regionally specified neural progenitors and functional neurons from human embryonic stem cells under defined conditions. Cell Rep. *1*, 703–714.

Kirkeby, A., and Parmar, M. (2012a). Building authentic midbrain dopaminergic neurons from

stem cells - lessons from development. Transl. Neurosci. *3*, 314–319.

Kirkeby, A., and Parmar, M. (2012b). Building authentic midbrain dopaminergic neurons from stem cells - lessons from development. Transl. Neurosci. *3*, 314–319.

Kobayashi, D., Kobayashi, M., Matsumoto, K., Ogura, T., Nakafuku, M., and Shimamura, K. (2002). Early subdivisions in the neural plate define distinct competence for inductive signals. Development *129*, 83–93.

Kouwenhoven, W.M., von Oerthel, L., and Smidt, M.P. (2017). Pitx3 and En1 determine the size and molecular programming of the dopaminergic neuronal pool. PLoS One *12*, e0182421.

Kriks, S., Shim, J.-W., Piao, J., Ganat, Y.M., Wakeman, D.R., Xie, Z., Carrillo-Reid, L., Auyeung, G., Antonacci, C., Buch, A., et al. (2011). Dopamine neurons derived from human ES cells efficiently engraft in animal models of Parkinson's disease. Nature *480*, 547–551.

Lehnen, D., Barral, S., Cardoso, T., Grealish, S., Heuer, A., Smiyakin, A., Kirkeby, A., Kollet, J., Cremer, H., Parmar, M., et al. (2017). IAP-based cell sorting results in homogeneous transplantable dopaminergic precursor cells derived from human pluripotent stem cells. Stem Cell Rep. *9*, 1207–1220.

Lindvall, O. (2012). Dopaminergic neurons for Parkinson's therapy. Nat. Biotechnol. *30*, 56–58.

McGrew, L.L., Lai, C.J., and Moon, R.T. (1995). Specification of the anteroposterior neural Axis through synergistic interaction of the Wnt signaling cascade withnogginandfollistatin. Dev. Biol. *172*, 337–342.

McKinney, W. (2010). Data structures for statistical computing in Python. In Proceedings of the 9th Python in Science Conference, S. van der Walt and J. Millman, eds., pp. 56–61.

Monzel, A.S., Smits, L.M., Hemmer, K., Hachi, S., Moreno, E.L., van Wuellen, T., Jarazo, J., Walter, J., Brüggemann, I., Boussaad, I., et al. (2017). Derivation of human midbrain-specific organoids from neuroepithelial stem cells. Stem Cell Rep. *8*, 1144–1154.

Müllner, D. (2011). Modern hierarchical, agglomerative clustering algorithms. arXiv, 1–29.

Nolbrant, S., Heuer, A., Parmar, M., and Kirkeby, A. (2017). Generation of high-purity human ventral midbrain dopaminergic progenitors for in vitro maturation and intracerebral transplantation. Nat. Protoc. *12*, 1962–1979.

Pandas Development Team, T. (2020). Pandas-Dev/Pandas: Pandas.

Peter, I.S., and Davidson, E.H. (2015). Genomic Control Process: Development and Evolution, Genomic Control Process: Development and Evolution (Elsevier Inc).

Peter, I.S., Faure, E., and Davidson, E.H. (2012). Predictive computation of genomic logic processing functions in embryonic development. Proc. Natl. Acad. Sci. U. S. A. *109*, 16434–16442.

Pöpperl, H., Schmidt, C., Wilson, V., Hume, C.R., Dodd, J., Krumlauf, R., and Beddington, R.S. (1997). Misexpression of Cwnt8C in the mouse induces an ectopic embryonic axis and causes a truncation of the anterior neuroectoderm. Development *124*, 2997–3005.

Prakash, N., Brodski, C., Naserke, T., Puelles, E., Gogoi, R., Hall, A., Panhuysen, M., Echevarria, D., Sussel, L., Vogt Weisenhorn, D.M., et al. (2006). A Wnt1-regulated genetic network controls the identity and fate of midbrain-dopaminergic progenitors in vivo. Development *133*, 89–98.

Puchkov, E. (2013). Intracellular viscosity: methods of measurement and role in metabolism. Biochem. Suppl. Ser. A. Membr. Cell Biol. *7*, 270–279.

Rifes, P., Isaksson, M., Rathore, G.S., Aldrin-Kirk, P., Møller, O.K., Barzaghi, G., Lee, J., Egerod, K.L., Rausch, D.M., Parmar, M., et al. (2020a). Modeling neural tube development by differentiation of human embryonic stem cells in a microfluidic WNT gradient. Nat. Biotechnol. *38*, 1265–1273.

Rifes, P., Isaksson, M., Rathore, G.S., Aldrin-Kirk, P., Møller, O.K., Barzaghi, G., Lee, J., Egerod, K.L., Rausch, D.M., Parmar, M., et al. (2020b). Modeling neural tube development by differentiation of human embryonic stem cells in a microfluidic WNT gradient. Nat. Biotechnol. 1–9.

Sagner, A., and Briscoe, J. (2017). Morphogen interpretation: concentration, time, competence, and signaling dynamics. Wiley Interdiscip. Rev. Dev. Biol. *6*, e271.

Scholpp, S., and Brand, M. (2003). Integrity of the midbrain region is required to maintain the diencephalic-mesencephalic boundary in zebrafishno isthmus/pax2.1 mutants. Dev. Dyn. *228*, 313–322.

Smits, L.M., Reinhardt, L., Reinhardt, P., Glatza, M., Monzel, A.S., Stanslowsky, N., Rosato-Siri, M.D., Zanon, A., Antony, P.M., Bellmann, J., et al. (2019). Modeling Parkinson's disease in midbrain-like organoids. NPJ Park. Dis. *5*, 5.

Stamos, J.L., and Weis, W.I. (2013). The -catenin destruction complex. Cold Spring Harb. Perspect. Biol. *5*, a007898.

Steinbeck, J.A., Choi, S.J., Mrejeru, A., Ganat, Y., Deisseroth, K., Sulzer, D., Mosharov, E.V., and Studer, L. (2015). Optogenetics enables functional analysis of human embryonic stem cell–derived grafts in a Parkinson's disease model. Nat. Biotechnol. *33*, 204–209.

Strano, A., Tuck, E., Stubbs, V.E., and Livesey, F.J. (2020). Variable outcomes in neural differentiation of human PSCs arise from intrinsic differences in developmental signaling pathways. Cell Rep. *31*, 107732.

Takagi, Y., Takahashi, J., Saiki, H., Morizane, A., Hayashi, T., Kishi, Y., Fukuda, H., Okamoto, Y., Koyanagi, M., Ideguchi, M., et al. (2005). Dopaminergic neurons generated from monkey embryonic stem cells function in a Parkinson primate model. J. Clin. Invest. *115*, 102–109.

Tao, Y., and Zhang, S.-C. (2016). Neural subtype specification from human pluripotent stem cells. Cell Stem Cell *19*, 573–586.

Toresson, H., Martinez-Barbera, J.P., Bardsley, A., Caubit, X., and Krauss, S. (1998). Conservation of BF-1 expression in amphioxus and zebrafish suggests evolutionary ancestry of anterior cell types that contribute to the vertebrate telencephalon. Dev. Genes Evol. *208*, 431–439.

Ulloa, F., and Martí, E. (2009). Wnt won the war: antagonistic role of Wnt over Shh controls dorso-ventral patterning of the vertebrate neural tube. Dev. Dyn. *239*, 69–76.

UniProt Consortium, T. (2018). UniProt: the universal protein knowledgebase. Nucleic Acids Res. *46*, 2699.

Virtanen, P., Gommers, R., Oliphant, T.E., Haberland, M., Reddy, T., Cournapeau, D., Burovski, E., Peterson, P., Weckesser, W., Bright, J., et al. (2020). SciPy 1.0: fundamental algorithms for scientific computing in Python. Nat. Methods *17*, 261–272.

Waskom, M. (2021). seaborn: statistical data visualization. J. Open Source Softw. *6*, 3021.

Wilson, L., and Maden, M. (2005). The mechanisms of dorsoventral patterning in the vertebrate neural tube. Dev. Biol. *282*, 1–13.

Xuan, S., Baptista, C.A., Balas, G., Tao, W., Soares, V.C., and Lai, E. (1995). Winged helix transcription factor BF-1 is essential for the development of the cerebral hemispheres. Neuron *14*, 1141–1152.

Zhou, W., Yui, M.A., Williams, B.A., Yun, J., Wold, B.J., Cai, L., and Rothenberg, E.V. (2019). Single-cell analysis reveals regulatory gene expression dynamics leading to lineage commitment in early T cell development. Cell Syst. *9*, 321–337.e9.

Q7 Q8

STAR★METHODS

KEY RESOURCES TABLE

REAGENT or RESOURCE	SOURCE	IDENTIFIER
Deposited data		
Raw data for optimisation of rostrocaudal model	(Kirkeby et al., 2012)	N/A
Processed data for optimisation of rostrocaudal model	This paper	https://github.com/max-brambach/neural_tube_patterning_paper
Data for validation of the 3D model	(Balaskas et al., 2012)	N/A
Software and algorithms		
Python 3.7	Python Software Foundation	https://www.python.org/
matplotlib 3.4.1	(Hunter, 2007)	https://matplotlib.org/
NumPy 1.20	(Harris et al., 2020)	https://numpy.org/
SciPy 1.4	(Virtanen et al., 2020)	https://www.scipy.org/
pandas 1.2.4	(McKinney, 2010; Pandas Development Team, 2020)	https://pandas.pydata.org/
seaborn 0.11.1	(Waskom, 2021)	https://seaborn.pydata.org/
MagicVoxel 0.98.2	ephtracy	https://ephtracy.github.io/
Analysis code	This paper	https://github.com/max-brambach/neural_tube_patterning_paper

RESOURCE AVAILABILITY

Lead contact

Further information and requests for resources and reagents should be directed to and will be fulfilled by the Lead Contact, Victor Olariu (victor@thep.lu.se).

Materials availability

This study did not generate new unique reagents.

Data and code availability

The used qPCR data and the code generated during this study are available at https://github.com/max-brambach/neural_tube_patterning_paper.

METHOD DETAILS

Clustering of gene expression data

For a gene with expression level x the normalisation was chosen such that $\overline{x} = \frac{x - x}{\sigma_x}$ with the normalised gene expression $\overline{x}$, the mean gene expression x across the different CT levels and the corresponding standard deviation σ_x.

The normalised gene expression data was clustered using hierarchical agglomerative clustering (Müllner, 2011). As method for the computation of the cluster distance, the Nearest Point Algorithm was used, meaning that the minimal distance between two clusters was considered as their distance. The used metric for the determination of the distance was the Euclidean distance.

Optimisation of model towards *in vitro* data

The parameters of the model were optimised using the experimental data. For the purpose of parameter optimisation, the expression of each brain region's specific genes was normalised such that its maximum value was 1 via division of all values by the maximum value. During the optimisation, the network was initialised with FB = MB = HB = GSK3 = 1 for all used CT concentrations. The evolution of the gene expressions was computed iteratively until the steady state was reached. This steady state gene expression for a specific

CT level was considered as the model output. The cost function E_d measures the deviation of the model output r from the data d and is computed using

$$E_d(p) = \sum_{CT}\sum_{i}(d_i - r_i(p))^2$$

with the first sum going over the different CT concentrations, the second sum going over $i \in \{FB, MB, HB\}$ and the parameter set $\boldsymbol{p}$. An optimal set of parameters is found by minimising $E_d(\boldsymbol{p})$ with respect to $\boldsymbol{p}$ using Q10 a L-BFGS-B algorithm (Byrd et al., 1995). The parameter set used in this article is shown in Table 1.

Similarity of model topologies

The similarity of different model topologies (Figure S1B) was quantified using a topology score defined as the number of identical interactions normalised to the total number of interactions of two model topologies. I.e. the topology score is zero for all interactions being different and one for identical topologies.

Differential equations

The set of differential equations describing the *in vitro* model mathematically was constructed using the Hill formalism and resulted in

$$\frac{d[FB]}{dt} = \frac{c_1[GSK3]^{n_1}}{1 + c_1[GSK3]^{n_1} + c_2[MB]^{n_2} + c_3[HB]^{n_3}} - \delta_1[FB]$$

$$\frac{d[MB]}{dt} = \frac{c_4[MB]^{n_4}}{1 + c_4[MB]^{n_4} + c_5[FB]^{n_5} + c_6[HB]^{n_6} + c_7[GSK3]^{n_7}} - \delta_2[MB]$$

$$\frac{d[HB]}{dt} = \frac{c_8[HB]^{n_8}}{1 + c_8[HB]^{n_8} + c_9[FB]^{n_9} + c_{10}[MB]^{n_{10}} + c_{11}[GSK3]^{n_{11}}} - \delta_3[HB]$$

$$\frac{d[GSK3]}{dt} = \frac{c_{12}[GSK3]^{n_{12}}}{1 + c_{12}[GSK3]^{n_{12}} + c_{13}[CT]^{n_{13}}} - \delta_4[GSK3]$$

$$\frac{d[CT]}{dt} = 0$$

The set of differential equations defining the *in vivo* model are

$$\frac{d[P]}{dt} = \frac{\alpha}{1 + \left(\frac{[N]}{N_{critP}}\right)^{h1} + \left(\frac{[O]}{O_{critP}}\right)^{h2}} - k_1[P]$$

$$\frac{d[O]}{dt} = \frac{\beta[G]}{1 + [G]} \cdot \frac{\alpha}{1 + \left(\frac{[N]}{N_{critO}}\right)^{h3}} - k_2[O]$$

$$\frac{d[N]}{dt} = \frac{\gamma[G]}{1 + [G]} \cdot \frac{1}{1 + \left(\frac{[O]}{O_{critN}}\right)^{h4} + \left(\frac{[P]}{P_{critN}}\right)^{h5}} - k_3[N]$$

$$\frac{d[GSK3]}{dt} = \frac{c_{12}[GSK3]^{n_{12}}}{1 + c_{12}[GSK3]^{n_{12}} + c_{13}[U]^{n_{13}}} - \delta_4[GSK3]$$

$$\frac{d[U]}{dt} = \frac{c_{14}[WNT]^{n_{14}} + c_{15}[U]^{n_{15}}}{1 + c_{14}[WNT]^{n_{14}} + c_{15}[U]^{n_{15}} + c_{16}[U]^{n_{16}}} - \delta_5[U]$$

$$\frac{d[G]}{dt} = \frac{\delta[SHH]}{1 + [SHH]} \cdot \frac{1}{1 + \left(\frac{[WNT]}{W_{critG}}\right)^{h6}} - k_4[G]$$

$$\frac{d[WNT]}{dt} = D_{WNT}\Delta[WNT] - \delta_{WNT}[WNT]$$

$$\frac{d[SHH]}{dt} = D_{SHH}\Delta[SHH] - \delta_{SHH}[SHH]$$

Please cite this article in press as: Brambach et al., Neural tube patterning: from a minimal model for rostrocaudal patterning toward an integrated 3D model, iScience (2021), https://doi.org/10.1016/j.isci.2021.102559

CellPress
OPEN ACCESS

The self-activation term of FB used during the model selection is not shown in the equations above, since it is not used after this step. The term was implemented in the ODE for FB expression following the Hill formalism, i.e. in the denominator for repression and in both numerator and denominator for activation.

The morphogen diffusion of the *in vivo* model was implemented using the discrete version of Fick's law

$$\frac{d[X_i]}{d[t]} = D \sum_{j}^{N_{neighbours}} [X_j] - [X_i]$$

with the index of the current cell i, the diffusion constant D, and $[X]$ being the concentration of either WNT or SHH. For each time step, the morphogen gradient pattern was updated prior to the intra-cellular network. The model reaches a steady state pattern, as shown in Figure 3C.

Model parameters and initial conditions:

The model parameters are summarized in the following table. Unlisted parameters of the dorsoventral model are listed in (Balaskas et al., 2012).During the model selection, the FB self-interaction parameters of the optimal topologies were $c_0 = 0.0005$ and $n_0 = 1$.

i	1	2	3	4	5	6	7	8
c_i	0.015	8	1000	0.2	0.5	0.201	0.0048	0.205
n_i	4	4	4	1	2	1	3	1
δ_i	0.169	0.169	0.171	0.171	0.1			
i	9	10	11	12	13	14	15	16
c_i	2	0.05	0.051	0.248	0.152	50	0.14	25
n_i	3	1	3	1	1	2.5	1	4
δ	δ_{WNT}	δ_{SHH}	D_{WNT}	D_{SHH}	W_{critG}	h6	k_4	
5.0	0.04	0.1	150.7	133.4	1.0	1.0	0.15	

For the *in vitro* simulations, the system was initialised with FB = MB = HB = GSK3 = 1. The 3D *in vivo* simulations were initialised with WNT = SHH = U = P = O = N = 0, FB = GSK3 = 1, MB = HB = 0.001, G = 3. Morphogen producing cells were held constant at $WNT_{prod} = 2$, $SHH_{prod} = 1$.

Kd and oex implementation

The simulation of knockdown and overexpression *in vitro* was achieved by fixing the respective kd/oex node's derivative to 0. The initial expression of the node then determined its behaviour, with kd being simulated by $[\,]_0 = 0$ and oex $[\,]_0 = 1$. The morphogen secretion overexpression *in vivo* was implemented by increasing the morphogen level of the secreting cells ten-fold. In all cases, the steady state expression pattern is considered the model output.

Morphogen diffusivity

The morphogens' diffusion coefficients D_{WNT}, D_{SHH} were estimated following a method proposed by He and Niemeyer based on the radius of gyration R_G of the protein (He and Niemeyer, 2003). R_G was estimated according to (Hong and Lei, 2009) for physiological conditions:

$$R_G \approx 3N^{\frac{2}{5}}\mathring{A}$$

with N being the protein chain length (number of amino acids). Inserting the protein lengths $N_{WNT} = 370$ and $N_{SHH} = 462$ (UniProt Consortium, 2018) yields $R_{WNT} \approx 32\mathring{A} = 3.2nm$ and $R_{SHH} \approx 35\mathring{A} = 3.5nm$.

Subsequently, we applied the estimate

$$D = \frac{6.85 \times 10^{-15} T}{\eta \cdot \sqrt{M^{1/3} \cdot R_G}}$$

with D in units of $m^2 \cdot s^{-1}$, temperature $T = 310K$, viscosity $\eta = 0.75$ cP (Puchkov, 2013) and the molecular Mass M ($M_{WNT} = 40.98kDa$, $M_{SHH} = 49.61kDa$) in kg $\cdot kmol^{-1}$, yielding

$D_{WNT} = 150.7\mu m^2 s^{-1}$ and $D_{SHH} = 133.4\mu m^2 s^{-1}$.

Quantification of gene expression

Gene expression on a tube slice was quantified by calculating percentage p_{gene} of the slice area where the gene expression is higher than a threshold T:

$$p_{gene} = \frac{A_{gene>T}}{A_{Total}}$$

where $A_{gene>T}$ is the area where the gene expression is larger than the threshold and A_{Total} is the area of the entire slice. Since strength of expression varies for the rostrocaudal and the dorsoventral network branch, the threshold was set to T=1 for the dorsoventral branch and to T=0 for the rostrocaudal branch.

Computational methods

The *in vitro* model equations were solved numerically using a fourth order Runge-Kutta algorithm with temporal step size $h = 2$. The equations of the *in vivo* model were solved using the explicit Euler method.

All computational work was performed in Python 3.7 with the extensions NumPy (Harris et al., 2020), SciPy (Virtanen et al., 2020), Matplotlib (Hunter, 2007), Pandas (McKinney, 2010; Pandas Development Team, 2020) and seaborn (Waskom, 2021). The neural tube model and the sources of morphogen secretion were set up in MagicVoxel, an open source voxel editor software. Cells were approximated as cubes with side length 10μm with the morphogen secretions sites positioned as illustrated in Figure 3B.

QUANTIFICATION AND STATISTICAL ANALYSIS

All quantification and analyses were performed as described in the methods details and computational analyses sections of the STAR Methods. Data are presented as mean ± standard deviation.

iScience, Volume ■ ■

Supplemental information

Neural tube patterning: from a minimal model for rostrocaudal patterning toward an integrated 3D model

Max Brambach, Ariane Ernst, Sara Nolbrant, Janelle Drouin-Ouellet, Agnete Kirkeby, Malin Parmar, and Victor Olariu

Figure S1. Performance of the rostrocaudal model topology, Related to Figure 1

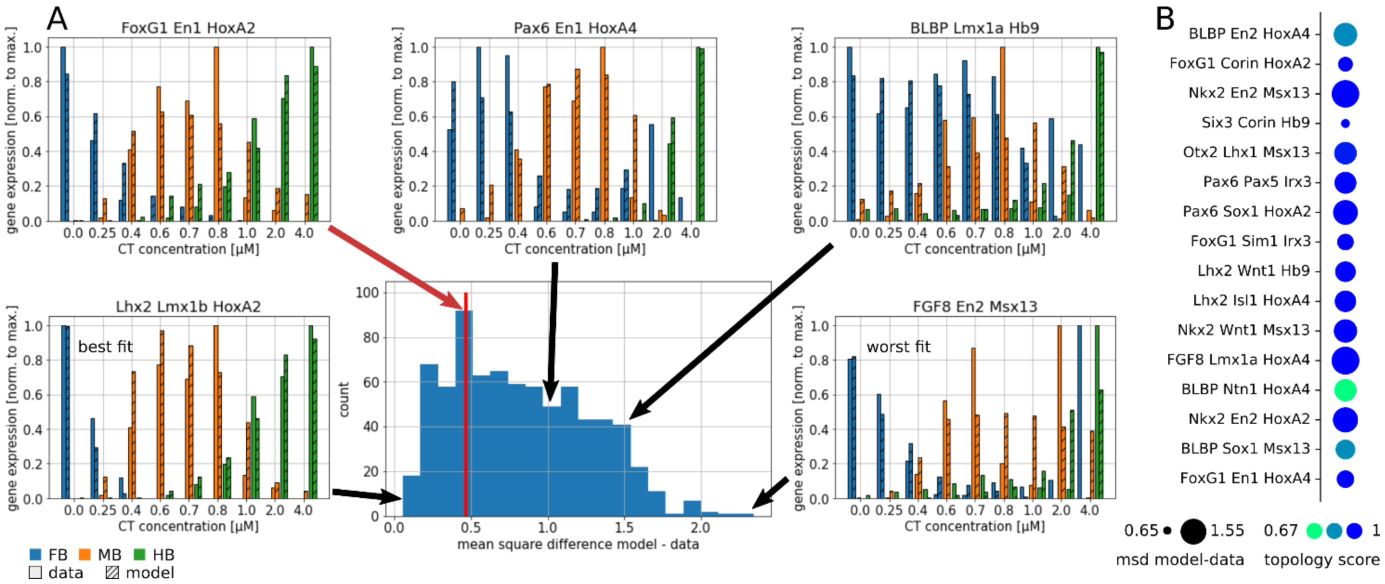

Figure S1: Performance of the rostrocaudal model topology. **A**: The rostrocaudal model was optimised towards all permutations of region-specific genes using the tristable topology (Figure 1 E). The histogram shows the distribution of mean squared differences (msd) between the data and the optimised model outputs. The red line indicates the gene selection used for further analysis (FoxG1, En1, HoxA2). The bar plots show examples of the detailed model performance for different msd values as indicated by the arrows. **B**: 16 gene combinations were randomly selected, and the optimal model topology was determined using the unbiased topology selection process. The similarity of the optimal model topology to the one found for (FoxG1, En1, HoxA2) is expressed using a topology score which is defined as the number of identical connections (activation or repression) normalised to the number of varied connections; 1=all connections are identical to the ones found for (FoxG1, EN1, HoxA2), 0=no connections match. Note that most optimal topologies are very similar or identical to the one used for (FoxG1, En1, HoxA2).

Figure S2. U-node parameter influence on patterning results, Related to Figure 3

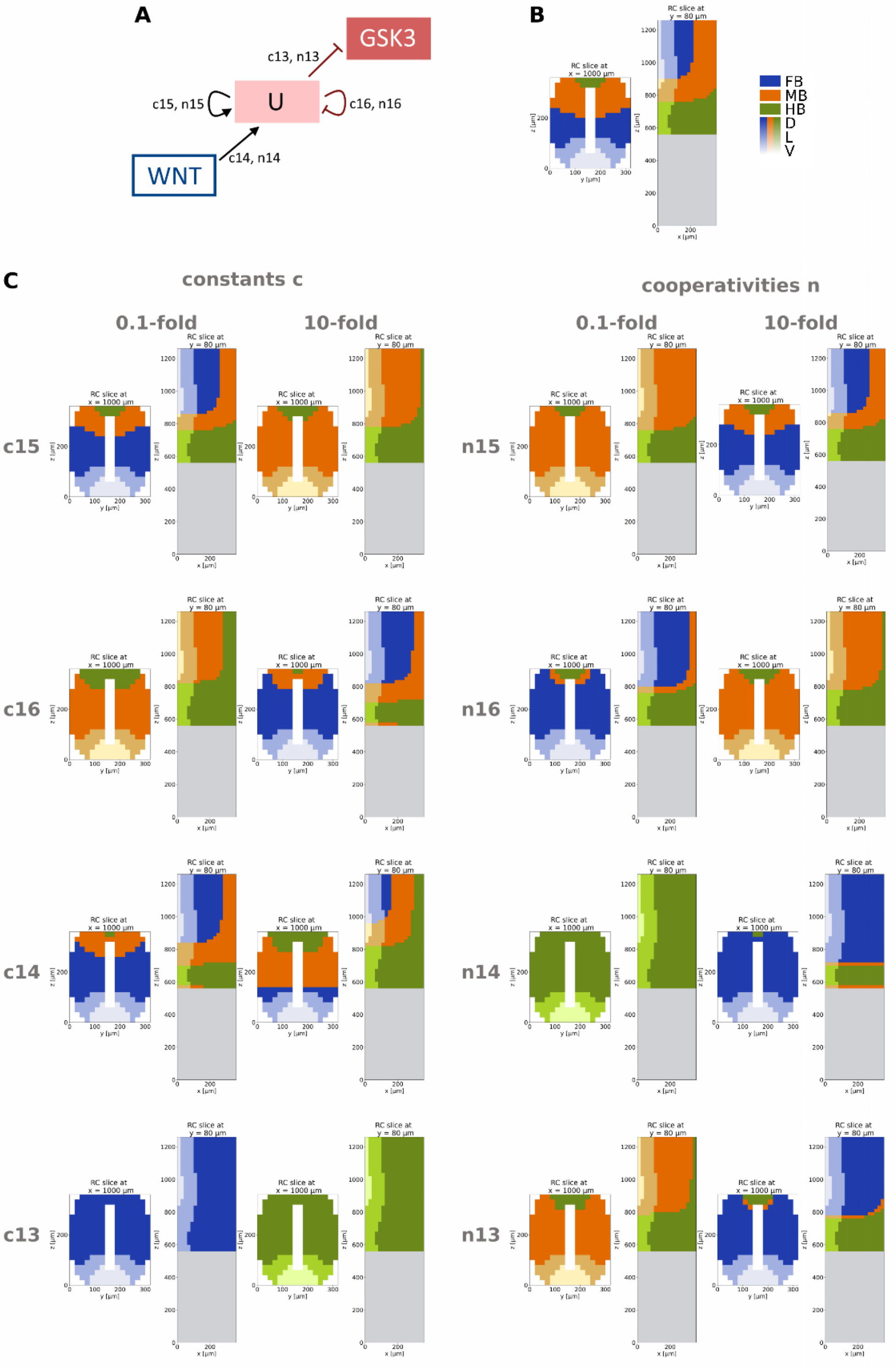

Figure S2: U-node parameter influence on patterning results. **A:** Illustration of the parameters that directly connected to the U-node levels; i.e. either targets or modulators. Shown are rate constants (c) and Hill coefficients (n). The numbers correspond to the parameters and equations in the Transparent Methods. **B:** Pattern resulting from the standard parameter set on the cylindrical tube (Supplementary Figure S3). **C:** Resulting patterns after scaling individual parameters 0.1-fold / 10-fold. Note that U self-activation /-repression (parameters 15, 16) exhibit converse sensitivity to parameter changes and primarily modulate the extent of the MB domain. Moreover, the pattern is less sensitive to the modulation of parameters associated with direct WNT than it is to the ones that act downstream repressing GSK3. This illustrates that the main function of U is to buffer the WNT signal for the rostrocaudal network branch.

Figure S3. Simplified model of the neural tube, Related to Figure 3

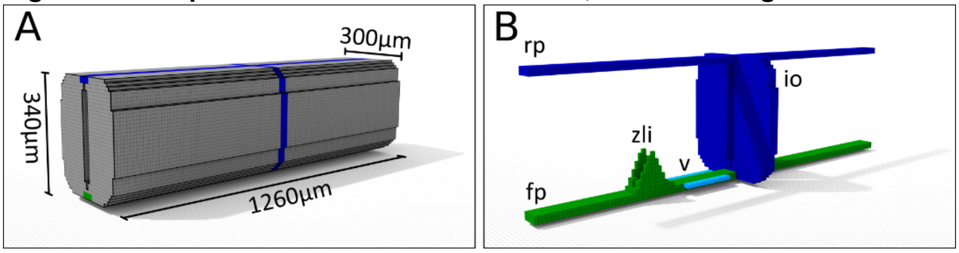

Figure S3: Simplification of neural tube geometry and important secretion points. **A:** Neural tube with secretion areas. **B:** Secretion areas including isthmic organizer (io), roof plate (rp), ventral midbrain (v), zona limitans (zli) and floor plate (fp).

Figure S4. Effect of tube geometry and secretion site setup on patterning, Related to Figure 3

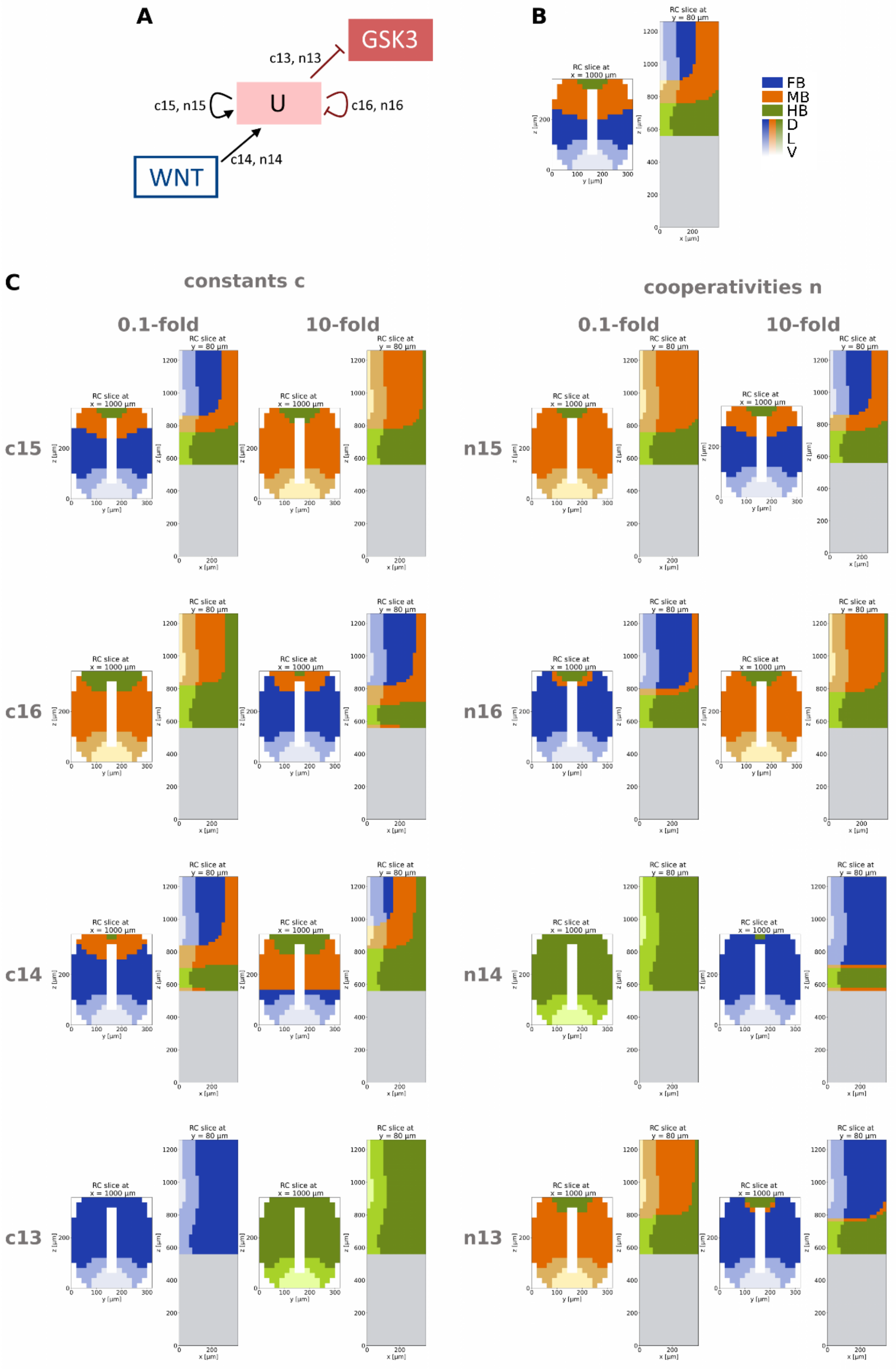

Figure S4: Effect of tube geometry and secretion site setup on patterning. The setup is indicated by the subtitle of each column (IO – isthmic organizer, ZLI – zona limitans, RP – roof plate, FP - floor plate). The top row shows patterning in the simplified tube model (Figure S2), while the bottom row displays the effect of the same setup for the realistic tube model (Figure 3b). Note that the roof plate (RP) signal strongly modulates the midbrain area and also causes stacking of the rostrocaudal brain.

Figure S5: SHH and WNT knockdown simulations, Related to Figure 5

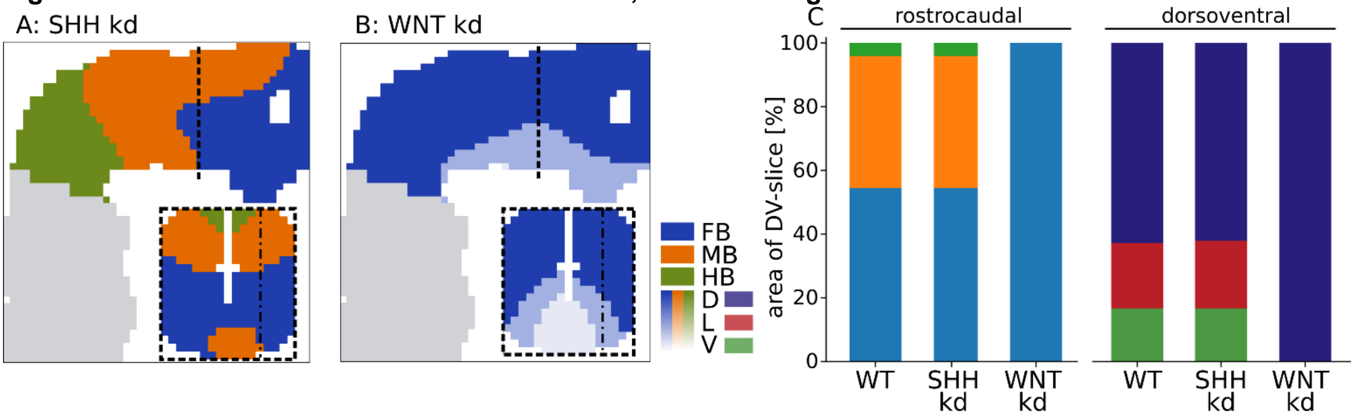

Figure S5: SHH and WNT knockdown simulations. **A:** Knockdown of SSH secretion. The rostrocaudal pattern was unaffected by the overexpression, whereas the dorsoventral pattern collapses and exhibits only D fate. **B:** Knockdown of WNT secretion. Predominantly, the rostrocaudal pattern was affected, which collapses and exhibits only FB fate. **C:** Quantification of the expression domain's sizes for the rostrocaudal and dorsoventral pattern analogous to Figure 4. Greyed areas are only shown for orientation and are not considered for pattern establishment. Dashed lines indicate the position of the transversal section (x = 580µm); dot-dashed lines indicate the position of the sagittal section (y = 80µm).
